# Signal transduction pathway mediated by the novel regulator LoiA for low oxygen tension induced *Salmonella* Typhimurium invasion

**DOI:** 10.1371/journal.ppat.1006429

**Published:** 2017-06-02

**Authors:** Lingyan Jiang, Lu Feng, Bin Yang, Wenwen Zhang, Peisheng Wang, Xiaohan Jiang, Lei Wang

**Affiliations:** 1 TEDA Institute of Biological Sciences and Biotechnology, Nankai University, Tianjin, P. R. China; 2 College of Environmental Science and Engineering, Nankai University, Tianjin, P. R. China; 3 The Key Laboratory of Molecular Microbiology and Technology, Ministry of Education, Tianjin, P. R. China; 4 Tianjin Key Laboratory of Microbial Functional Genomics, Tianjin, P. R. China; University of Illinois, UNITED STATES

## Abstract

*Salmonella enterica* serovar Typhimurium (*S*. Typhimurium) is a major intestinal pathogen of both humans and animals. *Salmonella* pathogenicity island 1 (SPI-1)-encoded virulence genes are required for *S*. Typhimurium invasion. While oxygen (O_2_) limitation is an important signal for SPI-1 induction under host conditions, how the signal is received and integrated to the central SPI-1 regulatory system in *S*. Typhimurium is not clear. Here, we report a signal transduction pathway that activates SPI-1 expression in response to low O_2_. A novel regulator encoded within SPI-14 (STM14_1008), named LoiA (low oxygen induced factor A), directly binds to the promoter and activates transcription of *hilD*, leading to the activation of *hilA* (the master activator of SPI-1). Deletion of *loiA* significantly decreased the transcription of *hilA*, *hilD* and other representative SPI-1 genes (*sipB*, *spaO*, *invH*, *prgH* and *invF*) under low O_2_ conditions. The response of LoiA to the low O_2_ signal is mediated by the ArcB/ArcA two-component system. Deletion of either *arcA* or *arcB* significantly decreased transcription of *loiA* under low O_2_ conditions. We also confirmed that SPI-14 contributes to *S*. Typhimurium virulence by affecting invasion, and that *loiA* is the virulence determinant of SPI-14. Mice infection assays showed that *S*. Typhimurium virulence was severely attenuated by deletion of either the entire SPI-14 region or the single *loiA* gene after oral infection, while the virulence was not affected by either deletion after intraperitoneal infection. The signal transduction pathway described represents an important mechanism for *S*. Typhimurium to sense and respond to low O_2_ conditions of the host intestinal tract for invasion. SPI-14-encoded *loiA* is an essential element of this pathway that integrates the low O_2_ signal into the SPI-1 regulatory system. Acquisition of SPI-14 is therefore crucial for the evolution of *S*. Typhimurium as an intestinal pathogen.

## Author summary

*Salmonella enterica* serovar Typhimurium (*S*. Typhimurium) is a major intestinal pathogen of both humans and animals. *Salmonella* pathogenicity island 1 (SPI-1) is required for host cell invasion by *S*. Typhimurium. Expression of SPI-1 genes is induced by low oxygen (O_2_) tension under host conditions, but the relevant regulatory mechanisms are not clear. Here, we report a low O_2_-induced signal transduction pathway for the activation of SPI-1 expression in *S*. Typhimurium. A novel regulator, STM14_1008 (named LoiA), encoded within SPI-14 directly activates *hilD*, which in turn activates *hilA* (the master activator of SPI-1), and thus other SPI-1 genes under O_2_-limited conditions. The response of LoiA to the low O_2_ signal is mediated by the ArcB/ArcA two-component system. We also confirmed that SPI-14 contributes to *S*. Typhimurium virulence by affecting invasion, with *loiA* as the virulence determinant. This novel SPI-1 activation pathway can be used by *S*. Typhimurium to sense and respond to low O_2_ conditions of the host intestinal tract for invasion. Acquisition of SPI-14 is therefore very important for the evolution of *S*. Typhimurium virulence by providing an essential component of this pathway, *loiA*.

## Introduction

*Salmonella* infects both humans and animals, resulting in a variety of diseases ranging from mild self-limiting gastroenteritis to severe systemic illness depending on serovar/host combination. *Salmonella enterica* serovar Typhimurium (*S*. Typhimurium) is a leading cause of human gastroenteritis, but can induce systemic disease in mice that resembles human typhoid fever [1,2]. *S*. Typhimurium pathogenicity and virulence mechanisms have been extensively studied using murine systems [1,3]. *S*. Typhimurium infection starts by ingestion of contaminated food or water. After surviving acidic challenge of the host stomach, *S*. Typhimurium enters the small intestine where it manages to invade and penetrate intestinal epithelium, leading to either inflammatory diarrhea confined to the intestinal tract or systemic spread of bacteria that are taken up by macrophages [3–5].

*Salmonella* pathogenesis is largely dependent on virulence genes encoded by *Salmonella* pathogenicity islands (SPIs). 23 SPIs have been identified so far, and 12 are present in *S*. Typhimurium (SPIs-1 to 6, 9, 11 to 14 and 16) [6,7]. SPI-1 and SPI-2, which encode separate type III secretion systems (T3SS-1 and T3SS-2, respectively) and effectors that are present in all *S*. *enterica* serovars, are the most important SPIs required for *Salmonella* pathogenicity. SPI-1 is required for *Salmonella* invasion into host intestinal epithelium, whereas SPI-2 is necessary for *Salmonella* survival and replication within macrophages and progress to systemic infection [1,8,9]. Virulence contributions of other SPIs in *S*. Typhimurium have also been investigated except for SPI-9 and SPI-14. SPI-3, SPI-6 and SPI-11 to 13 are involved in intracellular survival, contributing to *S*. Typhimurium systemic infection of mice [10–14]; SPI-4 and SPI-5 contribute to enteropathogenicity of *S*. Typhimurium by affecting bacterial adhesion or invasion, respectively [15,16]; SPI-16 is required for the long-term intestinal persistence of *S*. Typhimurium in mice [17]. Although not investigated in *S*. Typhimurium, SPI-9, encoding a type I secretion system, was reported to contribute to the adhesion of *S*. Typhi to epithelial cells [18], and biofilm formation and invasion in *S*. Enteritidis [19].

SPI-14 corresponds to a 9-kb region in *S*. Typhimurium [20]. It consists of eight open reading frames from STM14_1001 to STM14_1008 (S1 Fig), of which, *STM14_1008* encodes a putative LysR family transcriptional regulator, *STM14_1004* is a pseudogene and the other six encode putative cytoplasmic proteins of unknown function. The contribution of SPI-14 to *Salmonella* virulence has been investigated in *S*. Gallinarum and *S*. Enteritidis [20,21]. By using a PCR-based signature-tagged mutagenesis, two mutants of SPI-14 from *S*. Gallinarum (*SGA-8* and *SGC-8*, homologs of *S*. Typhimurium *STM14_1002* and *STM14_1008*, respectively) showed attenuated virulence in chickens [20]. How those two genes contribute to *S*. Gallinarum virulence was not investigated further. Using a similar approach, a mutant of SPI-14 from *S*. Enteritidis (*SEN0803*, homolog to *S*. Typhimurium *STM14_1005*) showed reduced invasion of Caco-2 cells and reduced invasiveness to chicken liver cells [21]. Therefore, it is most likely that SPI-14 also contributes to virulence in *S*. Typhimurium.

Invasion into host intestinal epithelial cells is the first crucial step in *S*. Typhimurium pathogenesis and is dependent on functions encoded by SPI-1 [1,2]. Effectors encoded within and outside SPI-1, and translocated by the SPI-1-encoded T3SS-1 into the intestinal epithelial cells of the eukaryotic host, are known to induce actin cytoskeletal rearrangements, leading to membrane ruffling and uptake of the invading *Salmonella* by the epithelial cells [3,22]. Expression of SPI-1 genes is induced when *S*. Typhimurium reaches the distal ileum of the host intestinal tract, the preferred invasion site, in response to combined environmental signals, including low O_2_, high osmolarity (salt), near neutral pH, and high acetate and high iron concentrations [23–26]. Low O_2_ tension is an important signal for invasion, as the expression of SPI-1 genes are highly induced under low O_2_ conditions [23,24,27]. However, regulatory mechanisms for low O_2_-induced SPI-1 activation are not clear.

A complex regulatory network has been reported for controlling SPI-1 expression. HilA encoded within SPI-1 is the master activator of SPI-1, which activates *prg* and *inv/spa* operons of SPI-1 directly and the *sip* operon of SPI-1 by acting through InvF, another SPI-1-encoded activator [28]. Expression of *hilA* is directly controlled by three AraC-like transcriptional regulators, including SPI-1-encoded HilC and HilD, and RtsA encoded outside of SPI-1, which constitute a feed-forward regulatory loop [29]. HilD is the dominant regulator of the system, as a *hilD* mutant had almost no *hilA* expression, whereas deletion of either *hilC* or *rtsA* decreased *hilA* expression less significantly [29,30]. Many other regulators encoded outside of SPI-1 affect SPI-1 genes and/or HilA expression through HilD, including HilE (repression of *hilA* by binding to and preventing HilD function) [31], FliZ (activation of *hilA* via post-translational regulation of HilD) [32], DNA adenine methylase (activation of *hilA* via post-translational regulation of HilD) [33], EnvZ-OmpR (activation of *hilA* via HilD) [34], CpxA/R (repression of *hilA* by decreasing the stability of HilD) [35], BarA/SirA (activation of *hilA* via activation of *csrB/csrC* to block CsrA repression of *hilD*) [24] and Fur (activation of *hilA* via an unknown regulation of HilD) [26]. In addition, Mlc, PhoP/Q, PhoB/R and FimZ/Y repress expression of SPI-1/HilA by acting through HilE [31,36,37].

The ability of *S*. Typhimurium to sense and respond to environmental signals at the distal ileum to induce SPI-1 expression is essential for invasion. Of the known regulators, the BarA/SirA two-component system mediates the response to high osmolarity for salt-induced SPI-1 activation while another osmo-regulator system, EnvZ/OmpR, is not involved [38]. SirA, independently of BarA, also activates *hilD* in response to the high acetate concentration (10–30 mM) found in the distal ileum [24]. As for low O_2_-induced SPI-1 activation, it was found that expression of *rtsA*, *hilC* and *hilD* genes is induced under low O_2_ conditions [39], indicating the involvement of those direct SPI-1 regulators. However, how this signal is received and integrated to the central SPI-1 regulatory system is not clear [25,39,40].

In this study, we investigated the role of SPI-14 in *S*. Typhimurium pathogenicity using the murine model. We found that deletion of SPI-14 significantly reduced invasion of *S*. Typhimurium into Caco-2 epithelial cells, and the BALB/c mice orally infected with the mutant contained fewer bacteria in the ileum, liver and spleen, and survived better, in comparison with the mice infected with the wild-type strain. Further mutation and complementation analysis revealed that the virulence determinant in SPI-14 is STM14_1008 which we named LoiA (low oxygen induced factor A), encoding a putative LysR family transcriptional regulator. Effects of LoiA on *S*. Typhimurium invasion were further investigated using *in vitro* and *in vivo* experiments. It was found that LoiA positively regulates the expression of *hilA* (the master activator of SPI-1) through direct activation of HilD under low O_2_ conditions, leading to the activation of SPI-1 genes for invasion. Finally, the response to low O_2_ conditions by LoiA was mediated by the ArcB/ArcA two-component regulatory system. Thus this study reports a novel low O_2_ signal transduction pathway, with SPI-14 encoded LoiA provided an essential element, for the activation of SPI-1 genes to facilitate *S*. Typhimurium invasion.

## Results

### SPI-14 deletion attenuated *S*. Typhimurium virulence in mice by affecting invasion

**Fig 1:**
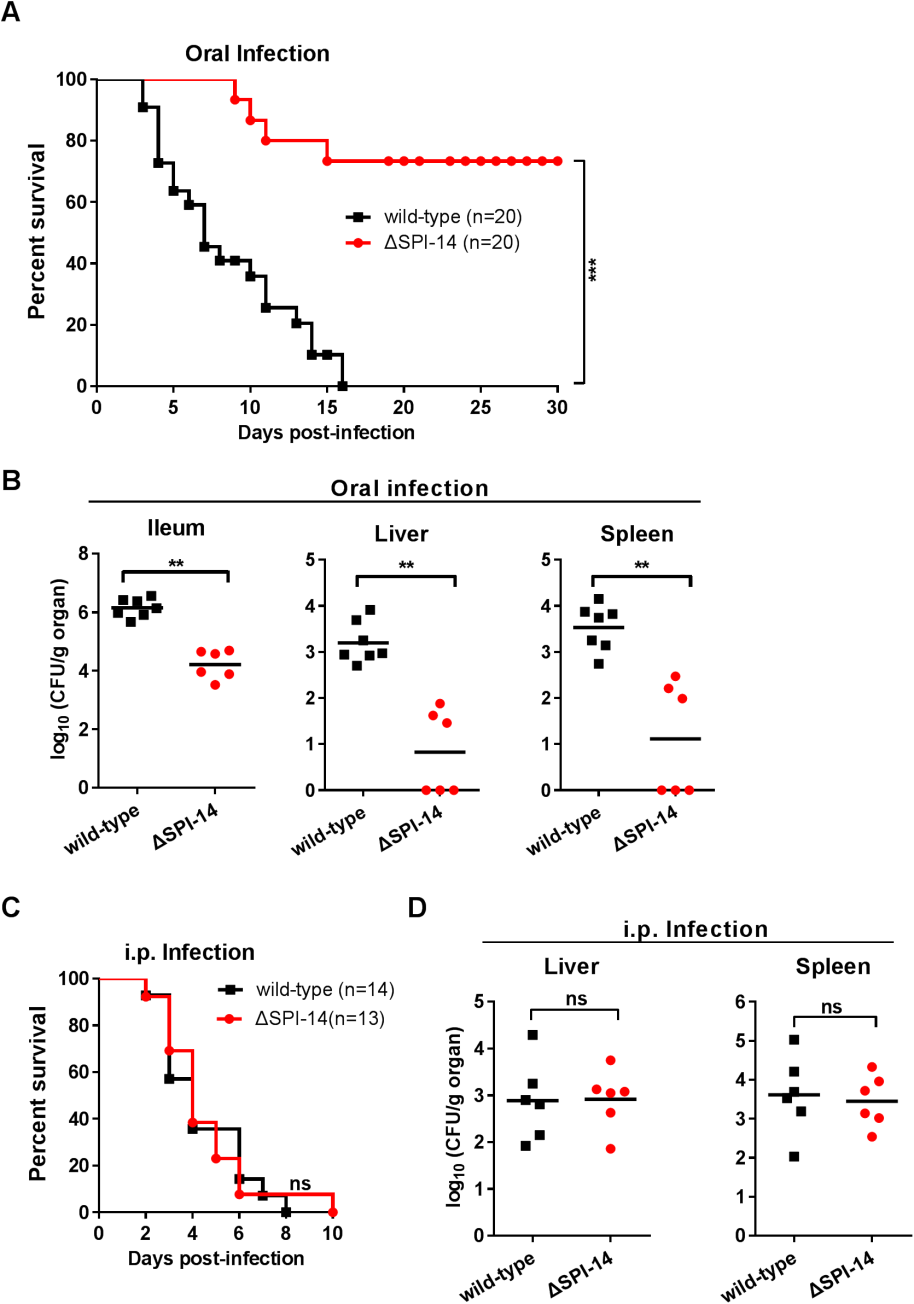
Lack of SPI-14 decreased *S*. Typhimurium virulence in mice by affecting intestinal invasion. (A) Survival plots of BALB/c mice for a 30-day period after orally infected with ~5×10^6^ CFU of wild-type or SPI-14 mutant. Data presented are the combination of three independent experiments, ^***^P<0.001 by log-rank curve comparison test. (B) Bacterial counts recovered from ileum, liver and spleen of the orally infected mice. At day 5 post-infection, mice organs were harvested and homogenized for colony enumeration. Data are combined from three independent experiments. Bars represent mean CFU of all mice, with *P* value determined by the Mann-Whitney U test (^**^P<0.01). (C) Survival plots of BALB/c mice after inoculation intraperitoneally (i.p.) with 1×10^4^ CFU of wild-type or SPI-14 mutant. Data presented are the combination of two independent experiments, with *P* value determined by log-rank curve comparison test (ns, not significant). (D) Bacterial counts recovered from liver and spleen of the i.p. infected mice. At day 3 post-infection, mice organs were harvested and homogenized for colony enumeration. Data are combined from two independent experiments. Bars represent mean CFU of all mice, with *P* value determined by the Mann-Whitney U test (ns, not significant).

To investigate the effect of SPI-14 in *S*. Typhimurium pathogenicity, the SPI-14 mutant of *S*. Typhimurium strain 14028 (wild-type) was generated. Two groups of 6- to 8-week-old female BALB/c mice were orally infected with approximately 5×10^6^ CFU of wild-type or SPI-14 mutant, respectively, and monitored for survival over a 30-day period. When challenged with the wild-type strain, the BALB/c mice started to die from day 3, and all had died within 16 days. In contrast, the mice infected with the SPI-14 mutant strain started to die from day 9, and only 25% had died after 16 days, with 75% surviving for the duration of the experiment (30 days) (Fig 1A). In addition, mice infected with the SPI-14 mutant had fewer bacteria in ileum (87-fold), livers (234-fold) and spleens (263-fold) than the mice infected with the wild-type strain, 5 days post-infection (Fig 1B). These results demonstrated that the deletion of SPI-14 severely attenuated the virulence of *S*. Typhimurium in mice after oral infection.

To determine whether the attenuated virulence caused by the deletion of SPI-14 in orally infected mice was due to its effect on invasion and/or systemic infection, we repeated mice infection assays by intraperitoneal (i.p.) injection, which allows *Salmonella* to directly disseminate to the systemic sites *via* the lymphatic and bloodstream system, bypassing the need for invasion of the intestine as in oral infection [41,42]. In contrast to the severely attenuated virulence of the SPI-14 mutant in orally infected mice, virulence in i.p. infected mice was not affected by deletion of SPI-14, as indicated by similar death rates and similar numbers of bacteria recovered from systemic organs (livers and spleens) between mice infected with the SPI-14 mutant and with the wild-type strain (Fig 1C and 1D). These results indicate that SPI-14 is not involved in systemic infection, and the attenuated virulence of the SPI-14 mutant observed in orally infected mice was likely due to the defect in intestinal invasion.

### SPI-14 deletion reduced *S*. Typhimurium invasion of epithelial cells

**Fig 2:**
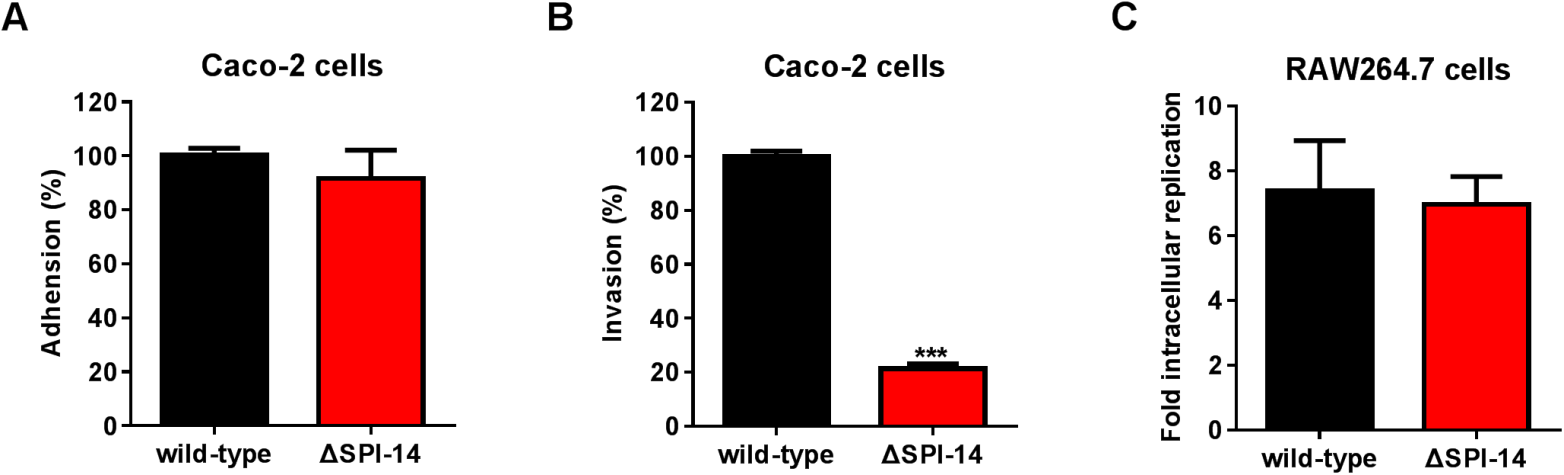
Lack of SPI-14 decreased *S*. Typhimurium invasion of Caco-2 epithelial cells. (A, B, C) Adhesion (A), invasion (B) and macrophage replication assays (C) of wild-type and SPI-14 mutant. Caco-2 cells or RAW264.7 cells were infected with wild-type *S*. Typhimurium or SPI-14 mutant at a multiplicity of infection (MOI) of 10. Adhesion and invasion ability of SPI-14 mutant were reported as percentage relative to the wild-type. Replication in RAW264.7 cells was determined by the ratio of the number of intracellular bacteria at 16 h post-infection to the number of bacteria at 2 h post-infection. Data are representative of at least three independent experiments and are presented as mean ± SD; *P* value was determined by student’s t test (^***^P<0.001).

How SPI-14 contributes to *S*. Typhimurium pathogenicity was further investigated by examining the effect of SPI-14 deletion on the ability of *S*. Typhimurium to adhere to and/or invade Caco-2 epithelial cells, and to replicate in murine RAW264.7 macrophages. As indicated by gentamicin protection assays, SPI-14 mutant and wild-type strains showed similar adherence ability to Caco-2 cells (Fig 2A); however, the invasion ability of the SPI-14 mutant to Caco-2 cells was decreased 4.8-fold compared with the wild-type strain (Fig 2B), indicating that SPI-14 contributes to *S*. Typhimurium invasion of, but not adherence to, epithelial cells. The replication fold in RAW264.7 macrophages 16 h post-infection was similar for the SPI-14 mutant and wild-type strains (Fig 2C), indicating that SPI-14 does not have a role in intracellular replication of *S*. Typhimurium within murine macrophages. The effect of SPI-14 deletion on *S*. Typhimurium invasion was further confirmed by immunofluorescence microscopy examination. By examining at least 50 Caco-2 cells for each strain in random fields, we found 64% of Caco-2 cells infected by the wild-type strain contained a single bacterium and 26% contained two or more bacteria, while 10% of the infected cells did not contain bacteria. In contrast, 70% of the Caco-2 cells infected by the SPI-14 mutant strain did not contain bacteria, while only 28% of cells contained one bacterium and 2% of cells contained two bacteria (S2A Fig). On average, Caco-2 cells infected with the wild-type strain contained 1.42 bacteria, while Caco-2 cells infected with the SPI-14 mutant contained 0.27 bacteria (S2B Fig). The wild-type and SPI-14 mutant exhibited similar growth rates *in vitro* (S3A and S3B Fig), indicating that the decreased ability of the SPI-14 mutant to invade Caco-2 cells was not due to a growth defect.

Collectively, this result demonstrates that deletion of SPI-14 attenuates *S*. Typhimurium virulence by decreasing bacterial invasion of epithelial cells.

### The gene *loiA* is the virulence determinant in SPI-14

**Fig 3:**
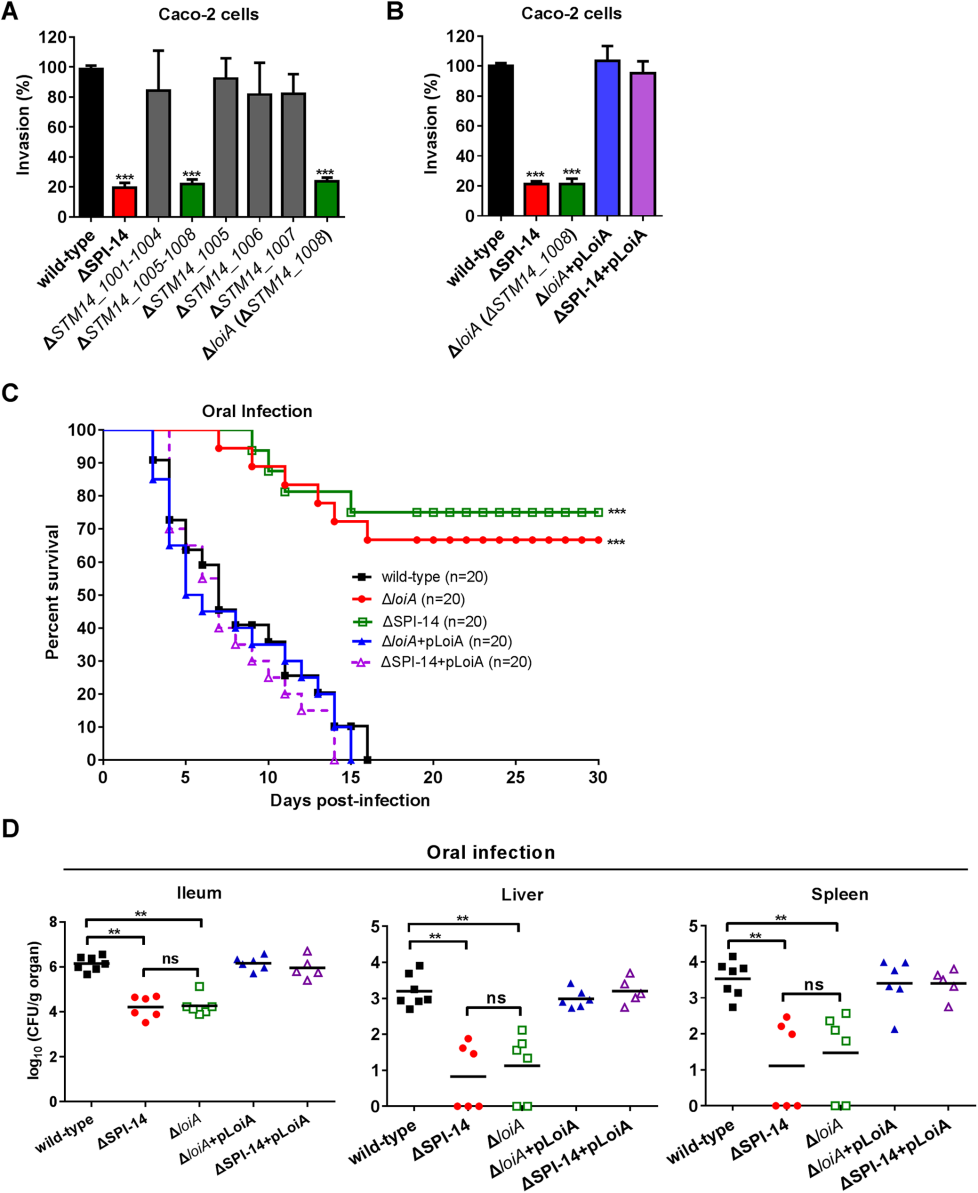
The gene *loiA* (*STM14_1008*) is the virulence determinant in SPI-14 influencing *S*. Typhimurium invasion. (A) Invasion assays of wild-type, *SPI-14* mutant, *STM14_1001-STM14_1004* mutant, *STM14_1005-STM14_1008* mutant, *STM14_1005* mutant, *STM14_1006* mutant, *STM14_1007* mutant and *loiA* (*STM14_1008*) mutant. (B) Invasion assays of wild-type, *SPI-14* mutant, *loiA* (*STM14_1008*) mutant and complemented strains. For (A) and (B), Caco-2 cells were infected with bacteria at an MOI of 10. The invasion ability of mutants is reported as percentages relative to the wild-type strain. Data are representative of at least three independent experiments and are presented as mean ±SD. *P* values were determined by student’s t test (^***^P<0.001). (C) Survival plots of BALB/c mice over a 30-day period after orally infected with ~5×10^6^ CFU of indicated bacterial strains. Data presented are the combination of three independent experiments, ^***^P<0.001 by log-rank curve comparison test. (D) Bacterial counts recovered from ileum, liver and spleen of the orally infected mice. At day 5 post-infection, mice organs were harvested and homogenized for colony enumeration. Data are combined from three independent experiments. Bars represent mean CFU of all mice, with *P* value determined by the Mann-Whitney U test (^**^P<0.01; ns, not significant).

To identify the virulence determinants in SPI-14 affecting *S*. Typhimurium invasion, mutant strains lacking the left (*STM14_1001* to *STM14_1004)* and right (*STM14_1005* to *STM14_1008)* regions of SPI-14 were generated and tested for invasion abilities into Caco-2 cells. Deletion of *STM14_1001* to *STM14_1004* had no effect on invasion, while deletion from *STM14_1005* to *STM14_1008* reduced bacterial invasion to the same level as the deletion of the entire SPI-14 region (Fig 3A). Mutants lacking each of the four genes from *STM14_1005* to *STM14_1008* were then generated and tested for invasion ability. Mutation in genes *STM14_1005*, *STM14_1006* and *STM14_1007* had no effect on invasion, while mutation in gene *STM14_1008* (named *loiA*) significantly reduced bacterial invasion (4.5-fold) (S2C Fig), similar to the level of reduction caused by the deletion of the entire SPI-14 region (4.8-fold) (Fig 3A and 3B). Mice infection assays showed that the survival rates of mice orally infected with the *loiA* mutant and the SPI-14 mutant were enhanced to similar levels, and the numbers of bacteria recovered from ileum, livers and spleens, respectively, were also reduced to similar levels between the two mutants, in comparison with the values obtained with the wild-type strain (Fig 3C and 3D). Complementation of either mutant by the expression of a functional *loiA* gene restored the virulence capacity of both *loiA* mutant and SPI-14 mutant strains to wild-type level (Fig 3C and 3D). Mice i.p. infection assays also found that the *loiA* mutant did not influence systemic infection (S4A and S4B Fig), in line with the result obtained with the SPI-14 mutant.

Collectively, these results demonstrate that the gene *loiA* is the virulence determinant in SPI-14 affecting *S*. Typhimurium invasion into epithelial cells.

### *loiA* deletion reduced the expression of *hilA* and SPI-1 genes

**Fig 4:**
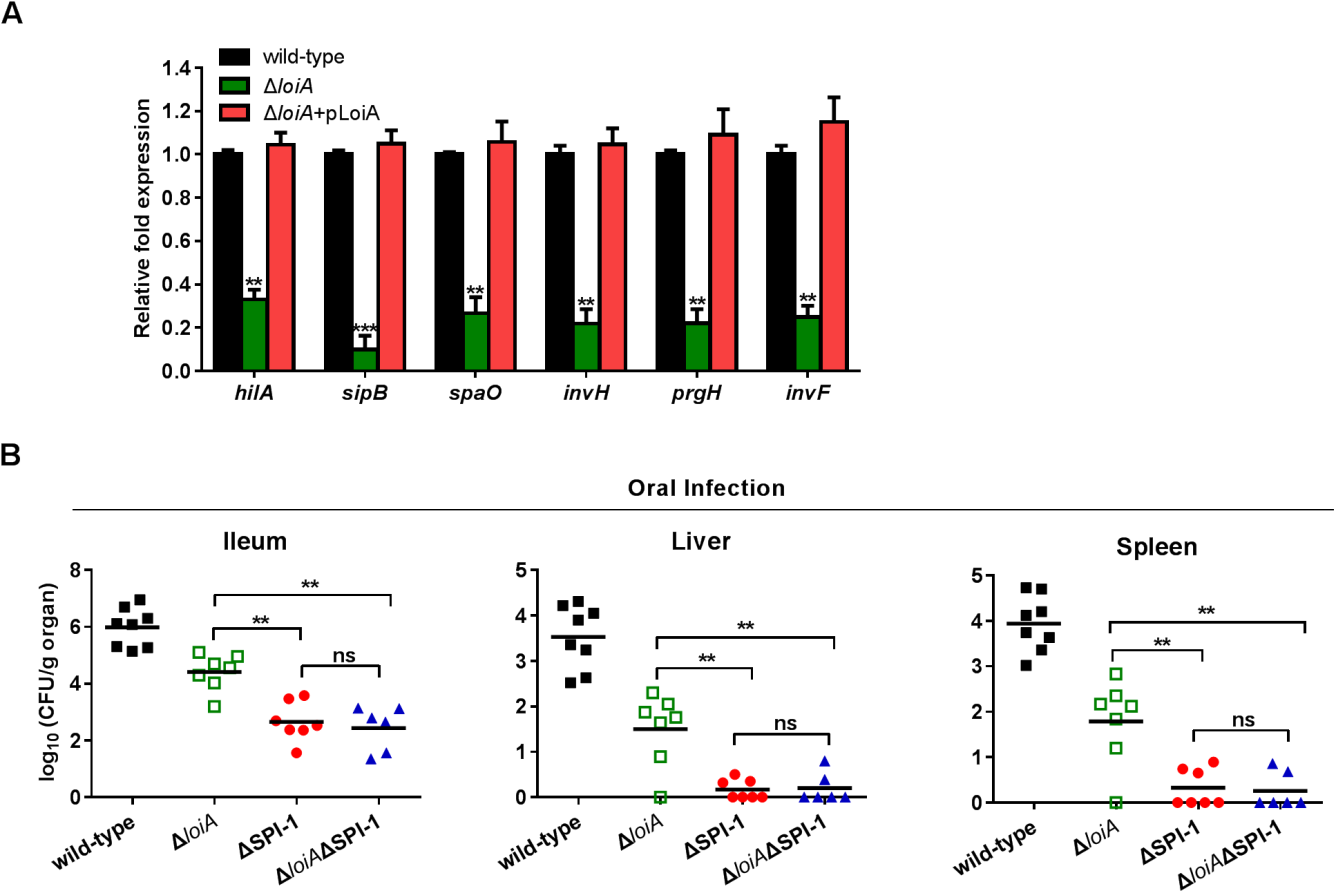
LoiA contributes to invasion through activating SPI-1. (A) Wild-type, *loiA* mutant and complemented strain were grown under SPI-1-inducing conditions (high salt, low O_2_) to late-exponential phase. Transcript levels of the SPI-1 genes in different strains were assessed by qRT-PCR. *16S rRNA* gene was used as the internal control. Data are representative of three independent experiments and are presented as mean ±SD. *P* values were determined by student’s t test (^**^P<0.01; ^***^ *P*<0.001). (B) Bacterial counts recovered from ileum, liver and spleen of the BALB/c mice orally infected with 5×10^6^ CFU of wild-type, *loiA* mutant, SPI-1 mutant or SPI-1/*loiA* double mutant at day 5 post-infection. Data are combined from three independent experiments. Bars represent mean CFU of all mice, with *P* values determined by the Mann-Whitney U test (^**^P<0.01; ns, not significant).

Considering the essential role of SPI-1 for *Salmonella* invasion, whether *loiA* has a role in regulating SPI-1 genes was investigated by quantitative real-time PCR (qRT-PCR) analysis. Expression of the SPI-1 master regulatory gene *hilA* and five HilA-regulated SPI-1 genes (*sipB*, *spaO*, *invH*, *prgH* and *invF*) was tested in the *loiA* mutant, in comparison with the wild-type strain, grown under SPI-1-inducing conditions (low O_2_, high salt). Expression of the five HilA-regulated SPI-1 genes was downregulated 4- to 10-fold in the mutant, while expression of *hilA* was downregulated 2.2-fold, in comparison with the expression of respective genes in the wild-type strain (Fig 4A). Complementation of a functional *loiA* gene to the *loiA* mutant restored the expression of all SPI-1 genes tested to wild-type levels (Fig 4A). These results indicate that LoiA functions as a positive regulator of *hilA* to activate SPI-1 genes.

To investigate whether LoiA affects invasion by regulating other virulence genes apart from SPI-1, a SPI-1 mutant (lacking the *sit* operon, which is required for systemic infection [43]) and a SPI-1/*loiA* double mutant were generated and used for mice infection assays. Mice orally infected with the SPI-1 mutant and SPI-1/*loiA* double mutant had similar numbers of bacteria in the organs tested (the ileums, livers and spleens), and the values from both mutant strains were significantly lower than those of *loiA* mutant infected mice (Fig 4B). These results indicate that the virulence defect of the SPI-1/*loiA* double mutant is caused by SPI-1 deletion, not *loiA* deletion, and thus the effect of *loiA* on virulence is due to its effect on SPI-1. In addition, through i.p infection of mice, we found that *loiA* did not confer an additional virulence defect by influencing systemic infection in a SPI-1 mutant background, since mice i.p. infected with the SPI-1 mutant or SPI-1/*loiA* double mutant also resulted in similar bacterial burdens in the livers and spleens (S5A and S5B Fig). Taken together, these findings confirmed that *loiA* affects *S*. Typhimurium virulence by affecting invasion via SPI-1.

### LoiA activates expression of *hilA* gene through activating *hilD*

**Fig 5:**
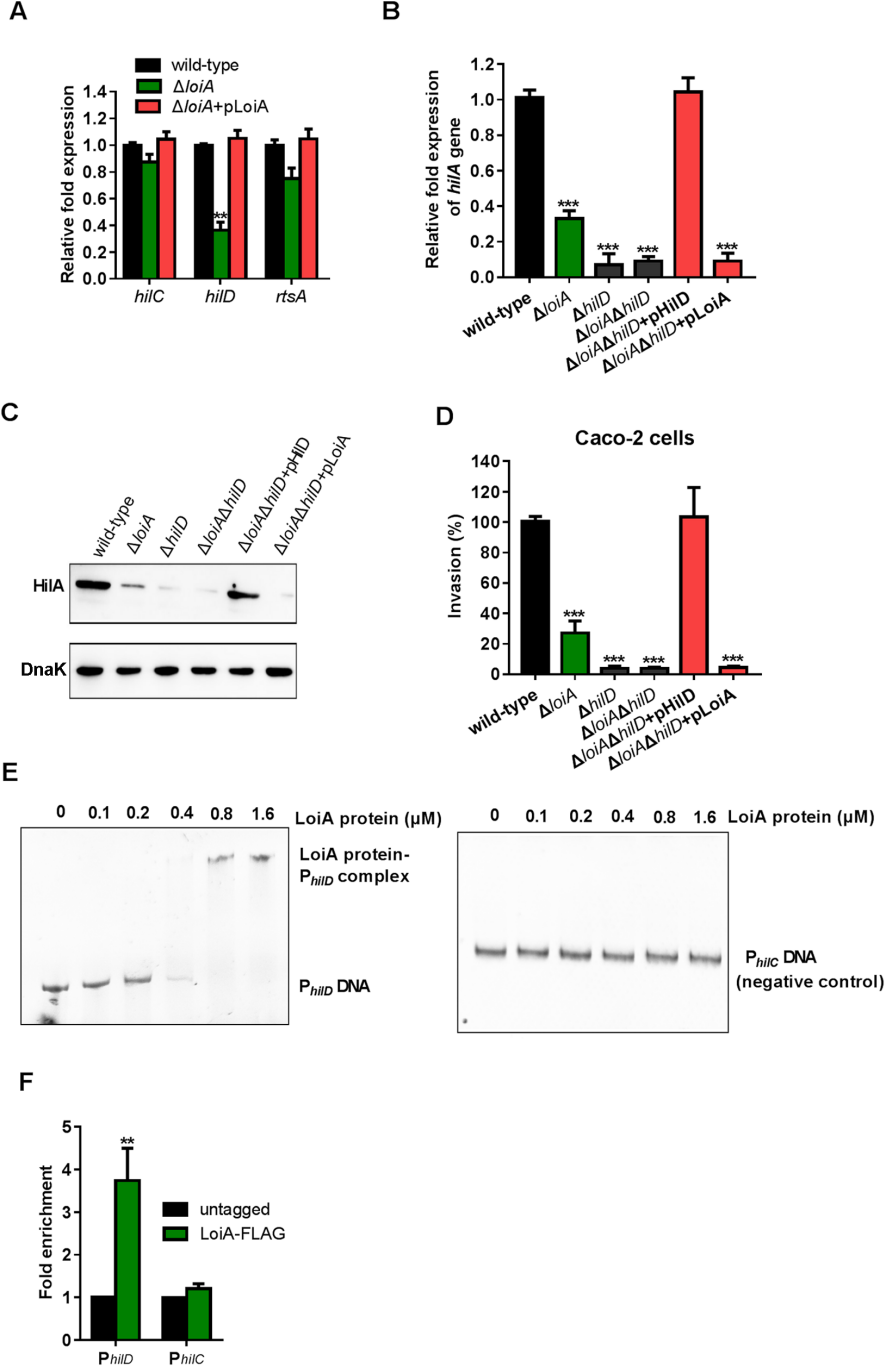
LoiA regulates *hilA* and other SPI-1 genes through HilD. (A) qRT-PCR analysis of changes in the expression of three direct higher regulators of *hilA* (*hilD*, *hilC*, and *rtsA*) in wild-type, Δ*loiA* and Δ*loiA*+pLoiA. Strains were grown under SPI-1-inducing conditions. (B) qRT-PCR analysis of changes in *hilA* expression in wild-type, Δ*loiA*, Δ*hilD*, Δ*loiA*Δ*hilD*, Δ*loiA*Δ*hilD*+pHilD and Δ*loiA*Δ*hilD*+pLoiA. Strains were grown under SPI-1-inducing conditions. (C) Western blot analysis of HilA in wild-type, Δ*loiA*, Δ*hilD*, Δ*loiA*Δ*hilD*, Δ*loiA*Δ*hilD*+pHilD and Δ*loiA*Δ*hilD*+pLoiA. Strains were grown under SPI-1-inducing conditions. Expression of the tagged HilA protein was determined using that of DnaK as the internal control. (D) Invasion assays of wild-type, Δ*loiA*, Δ*hilD*, Δ*loiA*Δ*hilD*, Δ*loiA*Δ*hilD*+pHilD and Δ*loiA*Δ*hilD*+pLoiA. Caco-2 cells were infected with bacteria at an MOI of 10. The invasion ability of mutants is reported as percentages relative to the wild-type strain. (E) EMSAs of *hilD* promoter DNA fragment with purified LoiA-His_6_ protein (0, 0.1, 0.2, 0.4, 0.8 and 1.6 μM). *hilC* promoter is used as a negative control. (F) Fold enrichment of the *hilD* promoter in ChIP samples, as measured *via* ChIP-qPCR. *hilC* promoter is used as negative control. Δ*loiA*, *loiA* mutant; Δ*loiA* +pLoiA, Δ*loiA* complemented strain; Δ*hilD*, *hilD* mutant; Δ*loiA*Δ*hilD*, *loiA*/*hilD* double mutant; Δ*loiA*Δ*hilD*+pHilD, *loiA*/*hilD* double mutant complemented with HilD; Δ*loiA*Δ*hilD*+pLoiA, *loiA*/*hilD* double mutant complemented with LoiA. Data are representative of at least three independent experiments and are presented as mean ±SD. *P* values were determined by student’s t test (^**^P<0.01; ^***^ *P*<0.001).

As the expression of *hilA* is directly controlled by HilD, HilC and RtsA, we tested whether LoiA regulates HilA through any of these three regulators. Using qRT-PCR analysis, *hilD* gene expression was significantly reduced in the *loiA* mutant compared with the wild-type strain *in vitro* under SPI-1-inducing conditions (2.4-fold) (Fig 5A), while the expression of *hilC* and *rtsA* in the *LoiA* mutant were not significantly affected (Fig 5A). These results indicate that LoiA regulates *hilD* positively, but has no direct effect on *hilC* or *rtsA*.

To further confirm that LoiA regulates *hilA* through *hilD*, a *hilD* mutant strain △*hilD*, the *loiA* and *hilD* double mutant strain Δ*loiA*Δ*hilD*, and the corresponding complemented strains Δ*loiA*Δ*hilD* +pHilD and Δ*loiA*Δ*hilD* +pLoiA, were constructed. Expression of *hilA* in the *loiA*/*hilD* double mutant was equivalent to that in the *hilD* mutant, with both mutants showing much lower levels of *hilA* expression than the reduced level in the *loiA* mutant, as evidenced by qRT-PCR analysis at the transcription level and western blotting at the protein level (Fig 5B and 5C), indicating that the presence of HilD is necessary for the activity of LoiA. The stronger reduction of *hilA* expression by the deletion of *hilD* is in line with the fact that *hilD* is regulated by many other regulators apart from LoiA. Complementation of the *loiA*/*hilD* double mutant with a functional *hilD* gene restored *hilA* expression, while complementation with a functional *loiA* gene could not restore *hilA* expression (Fig 5B and 5C). These results indicate that LoiA activates *hilA* via activating *hilD*. As expected, in invasion assays the *loiA*/*hilD* double mutant and the *hilD* mutant strains showed reduced invasion at similar levels, and complementation of the *loiA*/*hilD* double mutant with a functional *hilD* gene restored the invasion ability of this double mutant, while complementation of the double mutant with a functional *loiA* gene could not restore its invasion ability (Fig 5D). This indicates that LoiA activates invasion through *hilD*.

Electrophoretic mobility shift assays (EMSAs) were performed with purified LoiA-His_6_ protein to test whether LoiA directly binds the *hilD* promoter. As shown in Fig 5E, with increasing concentrations of LoiA protein, slowly migrating bands were observed for the *hilD* promoter, while no retarded bands were observed for *hilC* promoter (negative control), which indicated that LoiA can bind to the *hilD* promoter *in vitro*. Chromatin immunoprecipitation-quantitative PCR (ChIP-qPCR) analysis further demonstrated the binding of LoiA with the *hilD* promoter. The *hilD* promoter was exceedingly enriched in LoiA-ChIP samples, and the relative quantity was 3.8-fold higher than in the untagged control sample, while the control *hilC* promoter was not enriched in the LoiA-ChIP sample (Fig 5F).

Collectively, these results demonstrate that LoiA directly binds to the *hilD* promoter to activate *hilD* expression; and HilD in turn activates *hilA* expression, leading to the activation of SPI-1 genes.

### LoiA expression is activated under low O_2_ conditions

Signals for LoiA-induced SPI-1 activation were investigated. qRT-PCR analysis showed that *loiA* gene expression was 7.2-fold higher under the *in vitro* SPI-1-inducing conditions than under non-inducing conditions (low salt, high O_2_) (S6 Fig), indicating LoiA responds to high osmolarity or low O_2_ or both to regulate SPI-1 expression via HilD and HilA.

**Fig 6:**
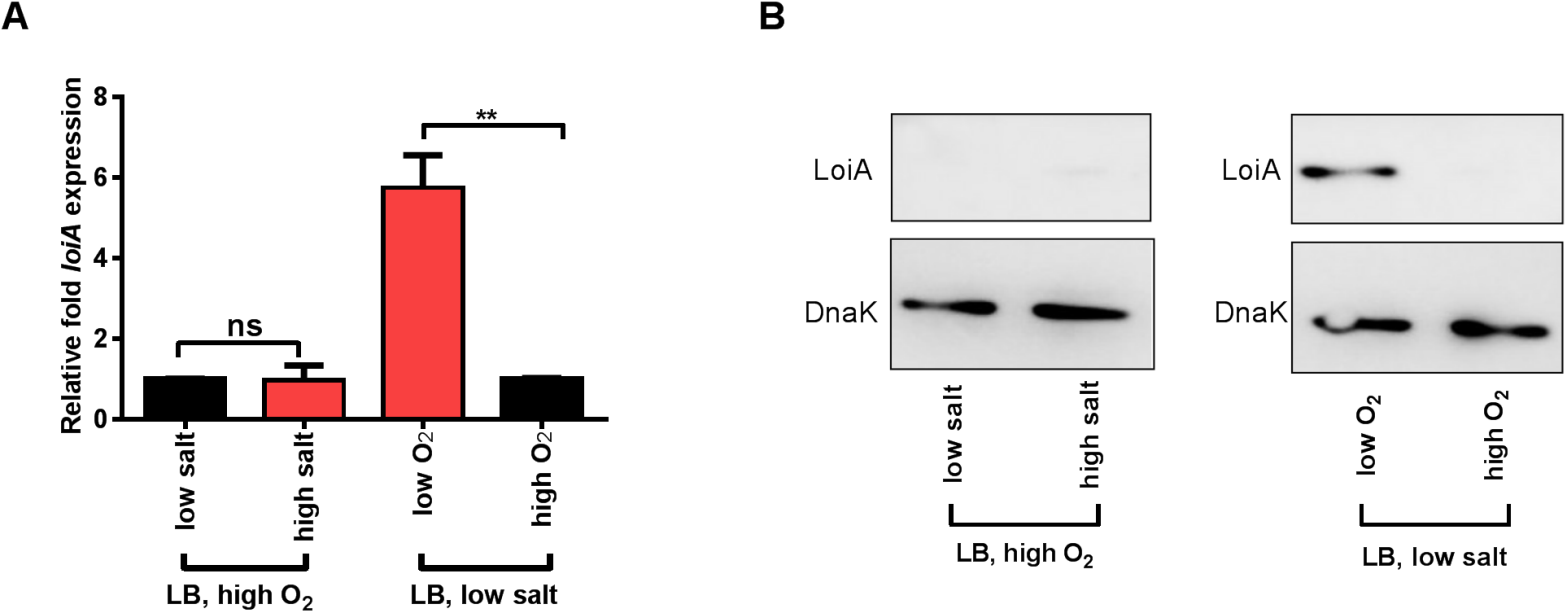
Expression of LoiA was induced by low O_2_ concentration. (A) qRT-PCR analysis of *loiA* gene expression under high O_2_ or low O_2_ (LB, low salt), and high salt (0.3M NaCl) or low salt (0.17M NaCl) conditions (LB, high O_2_). Data are representative of at least three independent experiments and are presented as mean ±SD. *P* values were determined by student’s t test (^**^P<0.01; ns, not significant). (B) Western blot analysis of LoiA protein level under high O_2_ or low O_2_ (LB, low salt), and high salt or low salt conditions (LB, high O_2_). The expression of the tagged LoiA protein was determined using that of DnaK as the internal control.

Further examinations revealed that *loiA* gene expression was 6.8-fold higher under low O_2_ and low salt conditions than under high O_2_ and low salt conditions in LB medium (0.17M NaCl) as indicated by qRT-PCR (Fig 6A) and western blot analysis (Fig 6B). In contrast, expression levels of LoiA were similar under both high salt (0.3M NaCl) and low salt (0.17M NaCl) conditions in the presence of a high level of O_2_ (Fig 6A and 6B). Thus, these results demonstrate that LoiA expression is activated by low O_2_ conditions, but not high osmolarity (high salt) conditions.

### Activation of LoiA expression in response to low O_2_ conditions is mediated by the ArcB/ArcA two-component regulatory system

Fnr (fumarate nitrate reduction regulator) and the ArcB/ArcA (aerobic respiratory control) two-component regulation system are well-known global regulators, which can sense and respond to changes in O_2_ availability and regulate gene expression in low O_2_ and anaerobic conditions. Whether LoiA responds to low O_2_ conditions through either of these two systems was investigated.

**Fig 7:**
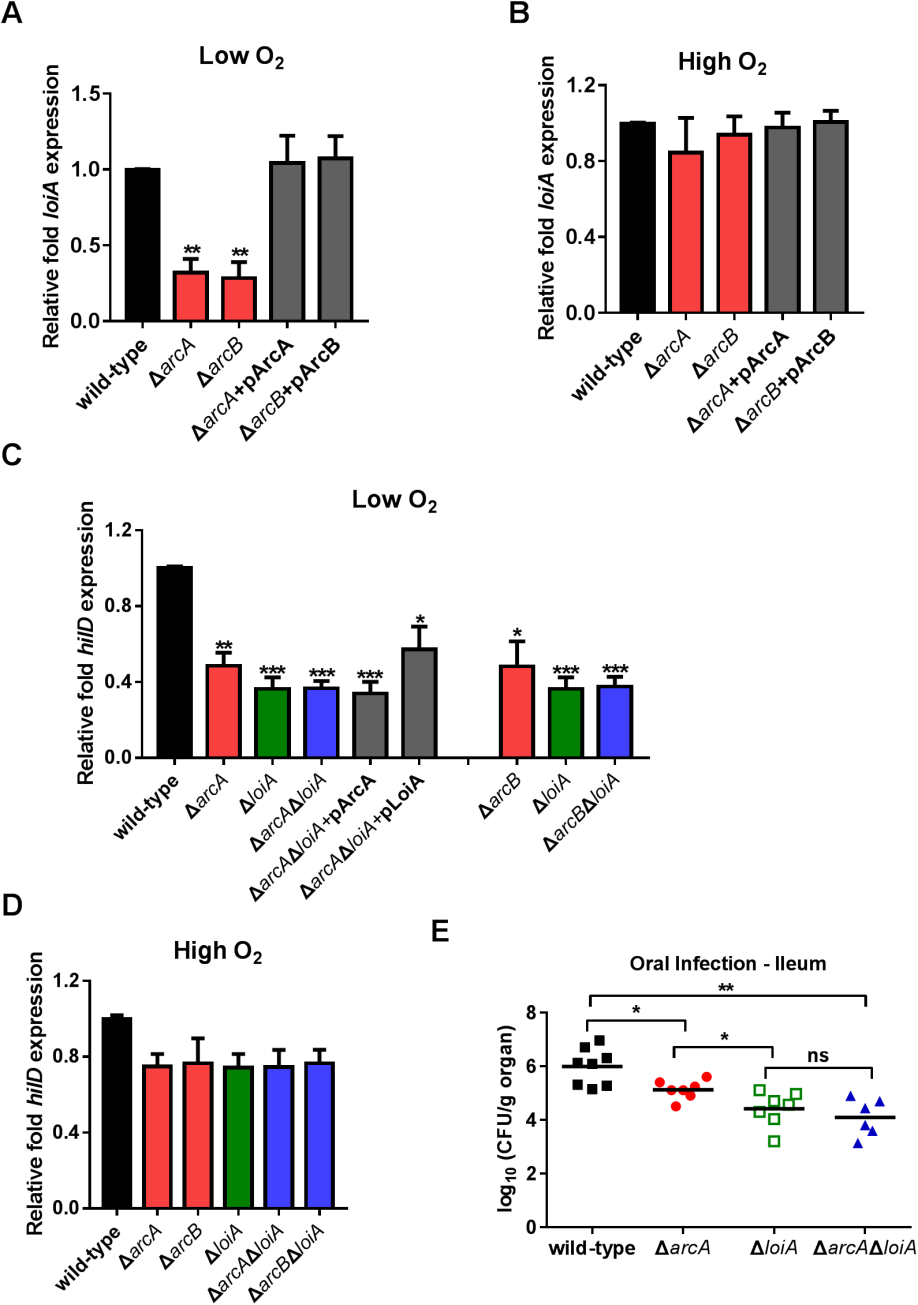
The activation of *LoiA* gene function by low O_2_ conditions is mediated by ArcAB. (A, B) qRT-PCR analysis of *loiA* gene expression in wild-type, *arcA* mutant, *arcB* mutant, and complemented strains for ArcA and ArcB. Bacteria were grown in LB medium (0.17 M NaCl) either with low O_2_ (A) or high O_2_ (B). (C, D) qRT-PCR analysis of *hilD* gene expression in wild-type, *arcA* mutant, *arcB* mutant, *loiA* mutant, *arcA*/*loiA* double mutant, *arcB*/*loiA* double mutant or complemented strains. Bacteria were grown in LB medium (0.17 M NaCl) either with low O_2_ (C) or high O_2_ (D). Data from graphs (A) to (D) are representative of at least three independent experiments and are presented as mean ±SD. *P* values were determined by student’s t test (^*^P<0.05; ^**^P<0.01). (E) Bacterial counts recovered from ileum of the BALB/c mice orally infected with 5×10^6^ CFU of wild-type, *loiA* mutant, *arcA* mutant or *arcA*/*loiA* double mutant at day 5 post-infection. Data are combined from three independent experiments. Bars represent mean CFU of all mice, with significance determined by the Mann-Whitney U test (^*^P<0.05; ^**^P<0.01; ns, not significant).

Mutant strains for *fnr*, *arcA* encoding the cognate response regulator and *arcB* encoding the membrane-bound sensor kinase were constructed, and expression levels of *loiA* in mutant strains were compared by qRT-PCR with that of the wild-type strain. The *fnr* mutant did not affect *loiA* gene expression in both low O_2_ and high O_2_ conditions (S7A and S7B Fig). Mutation of *arcA* or *arcB* significantly reduced *loiA* gene expression under low O_2_ conditions. Complementation of *arcA* and *arcB* mutants with the corresponding functional genes restored *loiA* gene expression to wild-type level (Fig 7A). Positive regulation of LoiA by the ArcB/ArcA system is dependent on low O_2_ conditions as the expression level of *loiA* in all mutant strains is comparable with the wild-type level under high O_2_ conditions (Fig 7B). These results showed that LoiA is able to sense and respond to low O_2_ via the ArcB/ArcA system for SPI-1 activation.

The connection between the ArcB/ArcA system and LoiA on SPI-1 regulation was further investigated by examing the expression levels of *hilD* in *arcA*, *arcB*, *loiA*, *arcA*/*loiA* and *arcB*/*loiA* double mutants grown under high and low O_2_ conditions. Expression of *hilD* was significantly reduced in all of the mutants when grown under low O_2_ conditions, in comparison to the wild-type strain (Fig 7C). However, the *loiA*, *arcA*/*loiA* and *arcB*/*loiA* double mutants showed similar levels of reduced *hilD* expression (2.8-, 2.9- and 2.7-fold, respectively), which were lower than the values of the *arcA* and *arcB* mutants (2.1- and 2.1-fold, respectively) (Fig 7C). Complementation of the *arcA*/*loiA* double mutant with LoiA restored *hilD* expression to the level of the *arcA* mutant, while there was no change in *hilD* expression level when complemented with *arcA* (Fig 7C). In addition, no significant reductions in *hilD* expression were detected in any of the tested mutants compared to the wild-type strain when bacteria were grown under high O_2_ conditions (Fig 7D). These results indicate that both ArcA and ArcB are involved in the regulation of *hilD* in response to low O_2_ conditions, and the ArcAB regulation on *hilD* is mediated by LoiA. Activation of the ArcAB system through LoiA during invasion was also confirmed by comparing the invasion phenotypes of the *loiA* mutant, the *arcA* mutant and *arcA*/*loiA* double mutant through orally infected mice. The bacterial burden in the ileum of *arcA*/*loiA* double mutant infected mice was equivalent to that of *loiA* mutant infected mice, and both were lower than that of *arcA* mutant infected mice (Fig 7E).

In addition, ArcA (the regulator gene of the ArcAB system) binding to the *loiA* promoter under low oxygen conditions was tested by EMSAs, with the ArcA-dependent *cydA* promoter used as a positive control [44,45]. Retardation of the *cydA* promoter (positive control) was clearly observed as the ArcA protein concentration increased, while no retardation could be detected of the *loiA* promoter (S8 Fig). This suggested that the positive regulation of ArcA on the *loiA* gene is indirect, with an unknown intermediate regulator.

## Discussion

**Fig 8:**
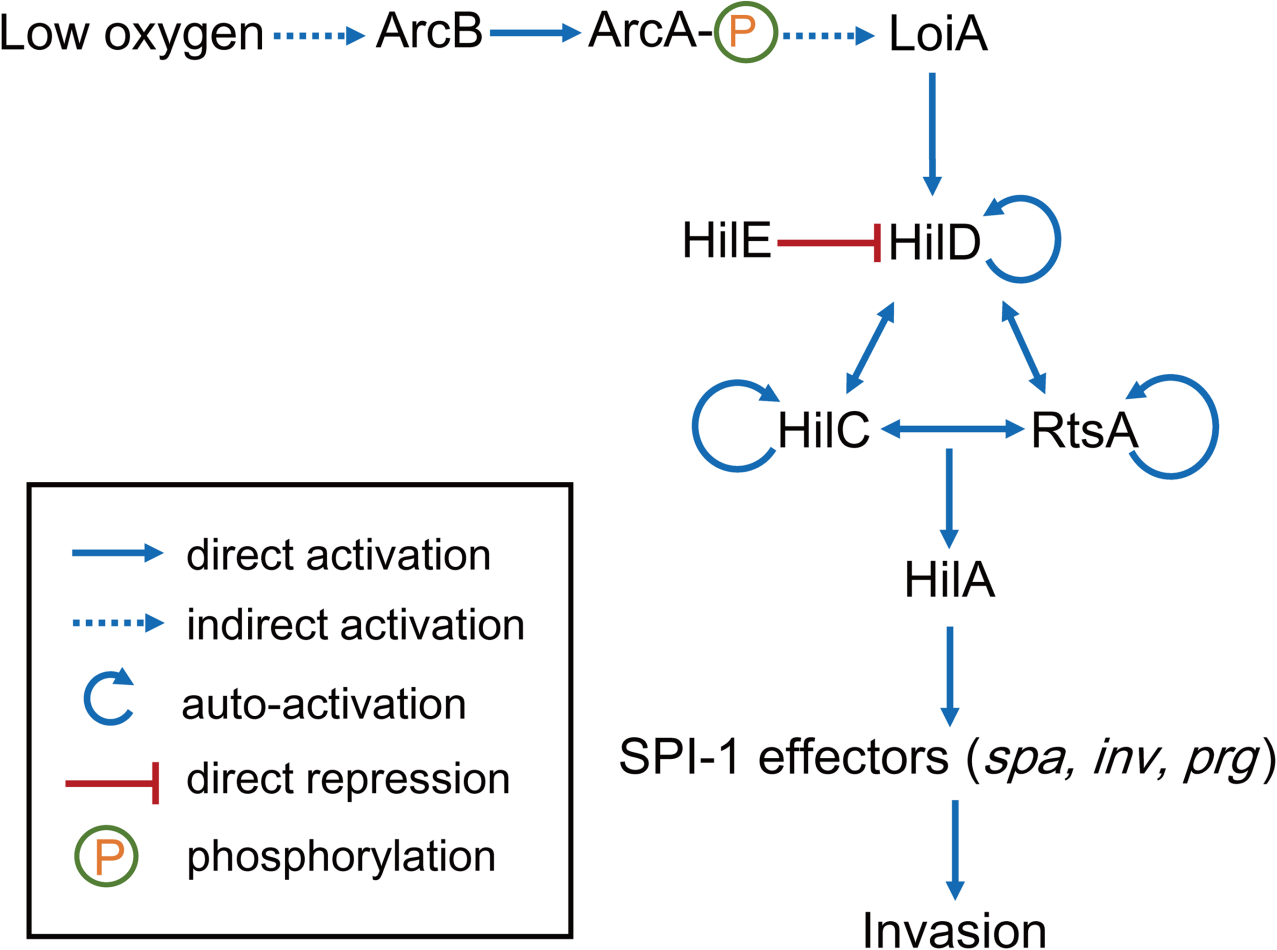
Model of the LoiA regulatory network in *S*. Typhimurium invasion. Under low O_2_ concentrations, the phosphorylated ArcA after sensing of low O_2_ signal by ArcB can indirectly activate *loiA* gene transcription, LoiA can then activate SPI-1 T3SS through directly activating HilD, thus promoting epithelial cell invasion.

In this study, we characterize and report a signal transduction regulatory pathway that *S*. Typhimurium uses to sense low O_2_ conditions of the host intestinal tract to activate expression of SPI-1 genes for invasion. A model for this regulatory pathway is proposed (Fig 8). Briefly, ArcB responds to low oxygen conditions when bacteria reach the distal ileum and undergoes autophosphorylation, following which the phosphate group is transferred to ArcA; phosphorylated ArcA (ArcA-P) activates *loiA* gene expression indirectly through unknown regulator(s); LoiA activates HilD directly through binding the *hilD* promoter; HilD then activates *hilA* and other SPI-1 genes, and thus facilitates the invasion process. Both SPI-1 expression and intestinal invasion ability was severely affected when the pathway was blocked by the deletion of *loiA*, indicating that the ability to sense and respond to low O_2_ using this pathway is very important for *S*. Typhimurium invasion, further confirming the signalling role of low O_2_ for SPI-1 induction. Clearly, LoiA plays an essential role by receiving the low O_2_ signal and passes it to the central SPI-1 regulation system (HilD). The production of HilD was suggested as an integration point for particular environmental signals and regulatory elements that then switches to turn on/off SPI-1 [46], and this also applied to the O_2_-responsive SPI-1 regulatory system described here. HilD, HilC and RtsA are known to form a feed-forward loop for SPI-1 regulation [29]. Therefore, regulation of one of those regulators is expected to have effects on the other two. However, significant reduction of *hilD* expression (2.4-fold) in a *loiA* mutant seemed to have little effect on *hilC* and *rtsA* expression (1.1- and 1.3-fold reduction, respectively). The lack of feed-forward effects of those three regulators were also reported previously [36,47]. While this cannot be explained clearly, one possibility is that HilD had a major effect on the regulation of downstream SPI-1 genes.

In addition to low O_2_ signal, the contributions of other site-specific signals or factors are necessary for *S*. Typhimurium to invade at the preferred site (ileum), as low O_2_ is a general condition of intestinal tracts of hosts. Several ileum-specific signals for SPI-1 activation have been reported including being rich in acetate and iron, near central pH, and low in concentration of propionate, butyrate and long-chain fatty acids, which can facilitate invasion at this site [23,24,26,48]. On the other hand, SPI-1 expression is repressed by the acidic pH in the stomach [27,49], by the high bile concentration in the proximal small intestine [50,51], and by the high concentration of long-chain fatty acids in the upper and middle sections of the small intestine [48], preventing invasion by *S*. Typhimurium at those sites prior to reaching the distal ileum. The concentration of those molecules decreased by absorption along the long length of the small intestine, and thus in the distal small intestine, the repression of SPI-1 may be relieved. In case of passing the preferred site of invasion, upon entering the large intestine, SPI-1 expression would be shut down by the increased concentration of propionate and butyrate synthesized by the resident microbiota [24,47]. Those hypotheses need to be confirmed experimentally in the future.

The ArcB/ArcA system is a well-known global regulator that controls expression of a large number of genes for growth under O_2_ limitation conditions, including genes for aerobic respiration and central metabolisms, as well as activation of the gene for the F-pilus [52,53]. Lim *et al*. also reported that deletion of *arcA* reduced *hilD* expression in *S*. Typhimurium strain SL1344 grown under aerobic conditions to early stationary phase, although no further information was given [54]. In this study, we clearly demonstrated the involvement of this system in a low O_2_-stimulated virulence regulation pathway. Activation of LoiA by the Arc system is indirect, and is mediated by unknown regulators that are under control of the Arc system, which remains to be investigated. Although the deletion of either the *arc* gene or *loiA* resulted in significantly decreased expression of *hilD* under low O_2_ conditions, the fact that *hilD* expression was still detected at lower levels (2.2- to 2.5-fold higher than that under high O_2_ conditions) (S9 Fig) indicated the presence of other low O_2_-signalling regulatory system(s) for SPI-1 activation.

Our study showed that Fnr, another well-known global regulator that senses and responds to O_2_ limitation, is not involved in the low O_2_-induced LoiA-mediated activation of SPI-1 genes as the expression of *loiA* was not affected by the deletion of *fnr*. Fnr has been previously reported to be a negative regulator of *hilA* in *S*. Typhimurium, as loss of Fnr caused a 1.5- to 2-fold increase of *hilA* expression under *in vitro* SPI-1-inducing conditions (low O_2_ and high salt) [46,55]. However, whether the repression of *hilA* by Fnr was induced by low O_2_ was not clarified in those studies. Furthermore, Fnr was reported not to influence low O_2_-mediated *Salmonella* invasion, as a *fnr* mutant did not differ in the ability to invade Madin-Darby canine kidney epithelial cells compared with wild-type *S*. Typhimurium, while both the *fnr* mutant and wild-type exhibited a significant increase in invasiveness when grown in low O_2_ *vs*. aerobic conditions [40]. On the other hand, Hassan *et al*. found that many SPI-1 genes (*prgKJIH*, *iagB*, *sicA*, *spaPO*, *invJICBAEGF*) had lower levels of expression in the *fnr* mutant than in wild-type *S*. Typhimurium (growing anaerobically) using microarray analysis [56]. Clearly, whether and how Fnr is involved in low O_2_-responsive SPI-1 regulation in *S*. Typhimurium need to be further clarified. Fur, the primary iron regulatory protein in *Salmonella*, can activate SPI-1 through HilD in response to the high concentration of Fe^2+^ in the small intestine [26]. Considering the low O_2_ conditions in the small intestine may contribute to the stability of Fe^2+^, whether low O_2_ indirectly influences Fur regulation on SPI-1 needs to be further investigated. Other unknown regulators may also be involved in low O_2_-mediated SPI-1 regulation. Taken together, these data and questions reflect the complicated nature of low O_2_-induced regulation of *Salmonella* invasion.

SPIs are regions of the bacterial chromosome harbouring virulence genes that are obtained *via* horizontal transmission from other bacteria at some point during evolution. Acquiring SPIs are considered to be ‘quantum leaps’ in *Salmonella* virulence evolution as critical virulence traits of *Salmonella* are directly linked to SPIs. SPI-1 is present in *Salmonella bongori* and all subspecies and serotypes of *S*. *enterica* analysed to date, and thus SPI-1 is suggested to be a rather ancient acquisition gained at the separation of the genera *Escherichia* and *Salmonella* from a common ancestor, and, relatively, the acquisition of other SPIs was later than that of SPI-1 [8]. Sequential acquisition of SPIs is important during *S*. Typhimurium evolution. While the acquisition of SPI-1 is a key event during the evolution of *Salmonella* virulence by providing the pathogen the ability for invasion, the acquisition of SPI-14 was crucial for the invasion to be induced under O_2_-limited conditions, in the host intestinal tract in this case, therefore representing another important event for the evolution of *S*. Typhimurium as an intestinal pathogen.

Genome analysis revealed that SPI-14 is present in most commonly reported non-typhoidal *S*. *enterica* serovars, including *S*. Typhimurium, *S*. Enteritidis, *S*. Gallinarum, *S*. Pullorum, *S*. Choleraesuis, and *S*. Dublin, while it is absent in the human-restricted serovars *S*. Typhi and *S*. Paratyphi A (the genome sequences of 104 representative *Salmonella enterica* strains for this analysis are available at ftp://ftp.ncbi.nlm.nih.gov/genomes/genbank/bacteria/Salmonella_enterica/). This indicates the presence of the LoiA-mediated low O_2_-responsive regulatory system in other SPI-14-containing serovars, and the absence of this pathway in the latter two serovars. The expression of SPI-1 genes and invasive phenotype of *S*. Paratyphi A are induced under low O_2_ conditions, but not under aerobic conditions [57], indicating that the bacterium uses other low O_2_-responsive regulatory mechanisms for the invasion of human intestinal epithelial cells. The same may apply to *S*. Typhi. Both await future studies. *SGA-8* (homolog to *STM14_1002*) and *SGC-8* (homolog to *loiA*) encoded by SPI-14 in *S*. Gallinarum and SEN0803 (homolog to *STM14_1005*) encoded by SPI-14 in *S*. Enteritidis were previously reported as virulence-related genes [20,21]. While *SGC-8* is expected to play the same role as *loiA* in *S*. Gallinarum, homologs of SGA-8 and SEN0803 (STM14_1002 and STM14_1005) were not related to virulence in *S*. Typhimurium. Due to the undefined role of SGA-8 in virulence [20], *STM14_1002* may contribute to virulence of *S*. Typhimurium in other ways, such as against the acid shock of the stomach, downregulation of inflammation after invasion and production of biofilm in the gall bladder to elicit chronic infections [1]; this needs further investigation. The contrary results for SEN0803 and STM14-1005 may be due to the different cell lines used for invasion assays (Caco-2 *vs*. LMH) and different hosts for virulence assays (chicken *vs*. mice), or a possibility of different functions or different regulation of this homologous gene in different serovars.

The novel low-O_2_ signal transduction pathway reported here constitutes both global regulators (ArcB/ArcA) and a specifically acquired regulator (LoiA), and direct regulators of SPI-1 (HilA, HilD), indicating the complex mechanisms of SPI-1 regulation. This study further enhanced our understanding on how *Salmonella* utilizes environmental cues to facilitate invasion.

## Materials and methods

### Ethics statement

All animal experiments were performed in accordance to the standards established in the Guide for the Care and Use of Laboratory Animals published by the Institute of Laboratory Animal Resources of the National Research Council (United States). The animal research procedures were approved by the Institutional Animal Care Committee at Nankai University and Tianjin Institute of Pharmaceutical Research New Drug Evaluation Co. Ltd (IACUC number: 2016032102), Tianjin, China. Every effort was made to minimize animal suffering and to reduce the number of animals used.

### Strains and plasmids

Bacterial strains and plasmids used in this study are listed in S1 Table. Oligonucleotides used in this study are listed in S2 Table. *S*. Typhimurium strain ATCC 14028 was used as wild-type strain throughout this study.

Mutant strains and the 3×FLAG-tagged strains were generated by the λ Red recombinase system as reported previously [58]. Briefly, PCR products for construction of mutants were generated from the chloramphenicol or kanamycin resistance genes of pKD3 or pKD4, respectively, using primers carrying at their 5’ ends 38–40 bp of homology to the regions flanking the start and stop codons of the gene to be deleted. Primers for 3×FLAG-tagged *loiA* or *hilA* alleles were designed to amplify the FLAG epitope coding sequence and chloramphenicol resistance gene using plasmid pWSK-FLAG (carrying 3×FLAG sequence and chloramphenicol resistance gene sequence) as the template. The resulting PCR products were electroporated into strain ATCC 14028 carrying the plasmid pKD46 for homologous recombination. The mutants and 3×FLAG-tagged bacteria undergoing homologous recombination were selected by their resistance to chloramphenicol or kanamycin and then verified by PCR amplification and sequencing. When required, the antibiotic resistance cassette was removed by FLP-mediated recombination with introduction of pCP20 plasmid [59].

Complementation mutants were generated by the expression of the corresponding functional genes (cloned from wild-type ATCC 14028) on a low-copy-number plasmid, pWSK129 [60]. To generate plasmids pLoiA, pHilD, pArcA and pArcB, the ORF and the upstream promoter sequence of the corresponding genes were amplified by PCR from genomic DNA of the wild-type strain. The resulting DNA fragment and pWSK129 vector were digested with the corresponding restriction enzymes (*Xba*I and *Bam*HI for construction of pLoiA and pHilD; *Bam*HI and *Eco*RI for construction of pArcB and pArcA). After DNA purification, the amplified PCR fragments were ligated into pWSK129 to give the recombinant plasmids and then transformed into corresponding mutant strains to give complemented strains. The pET-LoiA and pET-ArcA plasmids used for purification of the LoiA-His_6_ and ArcA-His_6_ proteins were generated by cloning the *loiA* and *arcA* gene sequence into the *Hin*dIII and *Bam*HI sites downstream of the His-tag element in plasmid pET-28a, respectively. All the resulting clones were verified by DNA sequencing.

### Bacterial growth conditions and cell culture

Bacteria were routinely grown in Luria–Bertani (LB) medium containing 1% tryptone, 0.5% yeast extract and 0.17 M NaCl at 37°C except for strains containing the temperature-sensitive plasmids pKD46 or pCP20, which were grown at 30°C. Under SPI-1-inducing conditions, bacteria were grown in 5 ml high salt LB medium (0.3 M NaCl); incubation was carried out in tightly closed 15 ml Falcon tubes without shaking (low O_2_). Under SPI-1 non-inducing conditions, bacteria were grown in 5 ml low salt LB medium (0.17 M NaCl); incubation was carried out in a 13 mm test tube with a loose cap with shaking at 200 rpm (high O_2_) [29,36,39]. Antibiotics were used at the following concentrations: ampicillin (Ap) 100 μg/ml, kanamycin (Km) 50 μg/ml, chloramphenicol (Cm) 20 μg/ml, streptomycin (Sm) 200 μg/ml and gentamicin (Gm) 10 or 100 μg/ml.

The human colon adenocarcinoma (Caco-2) cell line and murine macrophage cell line RAW264.7 were purchased from the Shanghai Institute of Biochemistry and Cell Biology of the Chinese Academy of Sciences (Shanghai, China). Cells were grown in RPMI-1640 medium (Gibco) supplemented with 10% fetal bovine serum (FBS, Gibco) and incubated at 37°C in 5% CO_2_. At 48 h before infection, cells were seeded into 12-well tissue culture plates, with or without coverslips, at the concentration of 1×10^5^ cells per well and maintained as differentiated monolayers.

### Mice infection

Laboratory animals Female BALB/c mice (6–8 weeks old) were purchased from Beijing Vital River Laboratory Animal Technology Co. Ltd (Beijing, China). All mice were maintained in a specific pathogen-free environment. Mice infections were performed as previously described [29,42]. Overnight bacterial cultures were 1:100 diluted and cultured under SPI-1-inducing conditions (high salt, low O_2_) to an OD_600_ of 0.6 (late-exponential phase). The collected bacteria were serially diluted to either 5×10^7^ CFU/ml in PBS (for oral infection) or 1×10^5^ CFU/ml in 0.9% NaCl (for i.p. infection). For oral infections, mice were given a single dose of 20 mg streptomycin 24 h prior to infection, followed by oral gavage with 5×10^6^ CFU of indicated *S*. Typhimurium strains in 0.1 ml PBS. For systemic infections, mice were given 1×10^4^ CFU of indicated *S*. Typhimurium strains in 0.1 ml 0.9% NaCl by i.p. injection.

The infected mice were monitored daily and the survival rates were recorded. For CFU enumeration experiments, the infected mice were euthanized 5 days post-infection (oral infection) or 3 days post-infection (i.p.). Ileum, spleen and liver were harvested, homogenized in PBS, diluted and then plated on LB plates for determination of CFU. Experiments were repeated two or three times.

### Adhesion and invasion assays

Adhesion and invasion assays were performed in the Caco-2 cell line as described previously with some modifications to the protocol [40,61]. Briefly, overnight bacteria were 1:100 sub-cultured into fresh LB medium (0.3 M NaCl) and incubated in tightly closed Falcon tubes without shaking until an OD_600_ of 0.6. Bacteria were pelleted, washed twice with PBS and re-suspended in RPMI medium. For the adhesion assays, both 12-well cell plates and bacterial suspensions were placed on ice for 15 min before infection. Ice-cold bacterial cells (500 μl per well) were added to Caco-2 cell monolayers at a multiplicity of infection (MOI) of 10 and incubated for 30 min on ice. For the invasion assays, bacterial cells were added to the Caco-2 cell monolayers at a MOI of 10 and incubated for 60 min at 37°C and 5% CO_2_. After washing three times with PBS, 500 μl RPMI medium containing 100 μg/ml gentamicin was added to the infected cells and incubated for an additional 60 min to kill extracellular bacteria. Following incubation, cells were washed three times with PBS, lysed with 500 μl 0.1% Triton X-100, and suitable dilutions were plated on LB agar plates containing appropriate antibiotics. Adhesion and invasion were calculated as percentages of the number of bacteria recovered from the total bacteria inoculated. All assays were performed with at least three independent biological replicates.

### Macrophage replication assays

The macrophage replication assays were conducted with stationary phase bacterial cells as reported previously [62]. Overnight bacteria were 1:100 sub-cultured into fresh LB medium (0.17 M NaCl) until an OD_600_ of 2.0. Bacteria were pelleted and opsonized in 10% normal mice serum for 20 min and then added to macrophage cell monolayers at an MOI of 10. The plates were centrifuged at 1,000g for 5 min to synchronize the infection. After incubation for 30 min at 37°C and 5% CO_2_, the infected cells were washed three times with PBS and incubated with medium containing 100 μg/ml gentamicin for 1 h, followed by medium containing 10 μg/ml gentamicin for the remaining time of infection. At 2 h and 16 h post-infection, the supernatant was removed and cells were washed three times with PBS and lysed with 0.1% Triton X-100. Serial dilutions of the lysates were plated onto LB agar to enumerate intracellular bacteria. The fold intracellular replication was calculated by dividing the intracellular bacterial load at 16 h by the bacterial load at 2 h. At least three independent biological replicates were performed.

### Immunofluorescence microscopy

For immunofluorescence microscopy, Caco-2 cells were seeded onto glass coverslips and infected as described above. After infection, cells were fixed with 3% paraformaldehyde (PFA) for 15 min at room temperature and washed three times with PBS. Cells were permeabilized for 20 min in 0.1% Triton X-100 and blocked with 5% BSA in PBS for 30 min. Mouse anti-*Salmonella* LPS (Abcam) was diluted 1:100 in PBS and applied for 1 h. Cells were washed three times with PBS, and then the secondary antibody goat anti-mouse IgG (FITC) (Abcam), diluted 1:200 in PBS, was applied for 1 h. Cells were washed again with PBS and incubated with DAPI (Invitrogen) for 2 min. After washing with PBS, cells were overlaid with 200 μl mounting medium. The cells were inspected for intracellular bacteria using a fluorescence microscope (Olympus) or a confocal laser scanning microscope (Leica) using filter sets for FITC (510 nm excitation, 530 nm emission). Images were further processed using the Leica TCS software package and Adobe Photoshop CS3.

### qRT-PCR

qRT-PCR was performed using the 7500 Real-Time PCR system (Applied Biosystems). To test the influence of *loiA* on expression of SPI-1 genes, the *loiA* mutant, complemented strain and wild-type strain were grown under SPI-1-inducing conditions for 4 h to late-exponential phase (OD_600_ ~0.6). To test changes in *hilA* expression in wild-type, Δ*loiA*, Δ*hilD*, Δ*loiA*Δ*hilD*, Δ*loiA*Δ*hilD*+pHilD and Δ*loiA*Δ*hilD*+pLoiA, strains were also grown under SPI-1-inducing conditions for 4 h to late-exponential phase (OD_600_ ~0.6). To test the influence of osmolarity and O_2_ level on *loiA* gene expression, the wild-type strain was grown under low O_2_, high O_2_, low osmolarity or high osmolarity conditions. For growth under high O_2_ and low O_2_ conditions, overnight bacterial cultures were 1:100 sub-cultured into fresh LB broth and then divided into two groups. One group was shaken at 200 rpm for an additional 4 h to late-exponential phase (OD_600_ ~1.2) with good aeration (high O_2_, control); the other group was transferred to a tightly closed Falcon tube and incubated standing for an additional 4 h to late-exponential phase (OD_600_ ~0.6; low O_2_). For growth under low osmolarity and high osmolarity conditions, overnight bacterial cultures were 1:100 sub-cultured into LB broth with 0.17 M NaCl (low salt) or 0.3 M NaCl (high salt) and shaken at 200 rpm for an additional 4 h. To test the influence of the *fnr* mutant and *arcA*/*arcB* mutant on *loiA* gene expression and the influence of the *arcA* or *arcB* mutants, and *arcA*/*loiA* or *arcB*/*loiA* double mutants on *hilD* gene expression, overnight bacterial cultures were sub-cultured 1:100 into fresh low-salt LB medium (0.17 M NaCl) and grown under high O_2_ or low O_2_ conditions for an additional 4 h to late-exponential phase (high O_2_: OD_600_ ~1.2; low O_2_: OD_600_ ~0.6).

Bacteria were pelleted by centrifugation, RNA samples were isolated using Trizol (Invitrogen), purified by the RNeasy Mini Kit (QIAGEN), DNase I treated (QIAGEN), reverse transcribed using random hexamers (Sigma) and processed for qRT-PCR. Each qRT-PCR reaction was carried out in a total volume of 20 μl in a 96-well optical reaction plate (Applied Biosystems) containing 10 μl FastStart Universal SYBR Green Master (ROX) mix, 1 μl cDNA, and two gene-specific primers with a final concentration of 0.3 mM each. The fold change in target gene relative to the housekeeping gene (*16S rRNA*) was determined by the 2^*-*ΔΔCt^ method. At least three biological replicates were performed for each qRT-PCR analysis.

### Expression and purification of LoiA-His_6_ and ArcA-his_6_

LoiA-His_6_ and ArcA-His_6_ fusion proteins were expressed in *Escherichia coli* BL21 containing pET-LoiA or pET-ArcA and purified from a soluble extract by using a HiTrap Ni^2+^-chelating column as previously described [63]. Protein concentration was determined by the Bradford procedure. Aliquots of the purified protein were stored at -70°C.

### Electrophoretic mobility shift assays (EMSAs)

EMSAs were performed as described previously with some modifications to the protocol [64,65]. PCR fragments encompassing the regulatory regions of *hilC*, *hilD*, *loiA* and *cydA* were amplified using genomic DNA of *S*. Typhimurium 14028 as a template. The DNA fragments were gel-purified. LoiA gel shift assays were performed by incubating the purified *hilD* and *hilC* promoter fragments (100 ng) at 37°C for 20 min with various concentrations of purified LoiA-His_6_ protein (0–1.6 μM) in a 20 μl solution containing the band-shift buffer (20 mM Tris-HCl (pH 7.5), 80 mM NaCl, 0.1 mM EDTA and 1 mM DTT). For ArcA O_2_ limitation gel shift assays, various concentrations of purified ArcA-His_6_ protein (0–1.6 μM) were incubated under low O_2_ for 20 min at 37°C with the amplified *loiA* and *cydA* promoter fragments (100 ng) in a 20 μl solution containing the above band-shift buffer. Samples were loaded with native binding buffer on a 6% polyacrylamide gel in 0.5× Tris-borate-EDTA. The DNA fragments were stained with ethidium bromide.

### ChIP-qPCR

The 3×FLAG-tagged strain (WT *loiA*-FLAG) was grown under SPI-1-inducing conditions and then pelleted by centrifugation. ChIP was performed based on established methods as reported previously [64,66]. Formaldehyde was added to bacterial cells (1% final concentration) for cross-linking and then incubated at room temperature for 25 min. Reactions were quenched with 0.5 M glycine, and samples were pelleted and washed three times with PBS. The samples were then used for ChIP following the Chromatin Immunoprecipitation kit (Millipore) protocol. The antibody used was the anti-FLAG mouse monoclonal antibody (Sigma). For ChIP-qPCR experiments, untreated chromatin was de-cross-linked by boiling for 10 min and purified for use as the “input” control. The relative enrichment of candidate gene promoters was performed with qRT-PCR and represents the value of the immunoprecipitated DNA divided by the input unprecipitated DNA. These values were normalized to the values obtained for each promoter precipitated using untagged wild-type in order to account for non-specific enrichment. The results represent the mean enrichment measured via qPCR in at least three biological replicate experiments.

### Western blot assays

To analyse HilA protein levels in wild-type, Δ*loiA*, Δ*hilD*, Δ*loiA*Δ*hilD*, Δ*loiA*Δ*hilD*+pHilD and Δ*loiA*Δ*hilD*+pLoiA strains, the corresponding 3×FLAG-tagged strains were grown in SPI-1-inducing conditions and then collected. To analyse the influence of osmolarity and O_2_ on the production of LoiA protein, the 3×FLAG-tagged strain (WT *loiA*-FLAG) was grown under the conditions indicated as described above (high O_2_ or low O_2_; high salt or low salt). Bacteria were pelleted by centrifugation, washed with PBS, resuspended in 100 μl SDS-polyacrylamide gel electrophoresis solubilization buffer (normalized for OD_600_ to ensure equivalent bacterial numbers) and lysed at 100°C for 10 min. Proteins were separated via 12% SDS-polyacrylamide gel electrophoresis and transferred to polyvinylidene difluoride membranes. The membranes were treated with 5% nonfat milk for 1 h to block non-specific binding and incubated with primary antibody raised in mice against the FLAG-tag (1:2,500 dilution, Sigma) or DnaK (1:5,000 dilution, Abcam) for 1 h, followed by washing in TBST. The blots were further incubated with the secondary antibody goat anti-mouse-HRP (1:5,000 dilution, CWBIO) for 1 h. Blots were washed in TBST followed by detection with the ECL enhanced chemiluminescence reagent.

### Statistical analysis

Statistical analysis was conducted using the software GraphPad Prism (v7.0). The mean ± SD from three independent experiments is shown in figures. Differences between two mean values were evaluated by two-tailed Student’s t test or Mann-Whitney U test. Multiple groups were compared by one-way ANOVA with Bonferroni’s post hoc analysis. To compare survival curves, the log-rank test was used. A *P* value <0.05 was considered to indicate statistical significance.

## Supporting information

S1 Fig Gene cluster of SPI-14 in *S.* Typhimurium strain 14028, with *STM14_1008* gene named *loiA* (low oxygen induced factor A). (TIF)

S2 Fig Immunofluorescence analysis showed the invasion defect of SPI-14 mutant and *loiA* mutant to Caco-2 cells. (A) The percentages of infected cells containing 0, 1, 2 or >2 bacteria. Caco-2 cells were seeded on coverslips and infected with bacteria at an MOI of 10. Infected cells were fixed 1 h post-infection and stained for immunofluorescence microscopy. The number of bacteria per Caco-2 cell was counted for at least 50 cells. Data are representative of at least three independent experiments and are presented as mean ±SD. (B) Number of intracellular bacteria per Caco-2 cell. Intracellular bacteria per cell were counted in random fields at 1 h post-infection. Bars show the mean number of bacteria contained in the infected Caco-2 cells that were counted. Data are representative of three independent experiments, with *P* values determined by student’s t test (^***^P<0.001; ns, not significant). (C) Representative images of infected Caco-2 cells by wild-type strain and *loiA* mutant. Bacteria were labelled with anti-*Salmonella* LPS antibody (green), and cell nuclei were counterstained with DAPI (blue).
(TIF)

S3 Fig Growth curves of *S.* Typhimurium strains *in vitro*. (A, B) Overnight culture of wild-type, SPI-14 mutant or *loiA* mutant were sub-cultured 1:100 into fresh high salt (0.3 M NaCl) LB medium and cultured for additional 24 h at 37°C with high O_2_ (A) or low O_2_ concentrations (B). The absorbance at 600 nm (OD_600_) of 2 ml aliquots of culture was measured regularly over this period. Data are representative of at least three independent experiments and are presented as mean ±SD.
(TIF)

S4 Fig Lack of *loiA* did not influence *S.* Typhimurium systemic infection of BALB/c mice. (A) Survival plots of BALB/c mice after inoculation intraperitoneally (i.p.) with 1×10^4^ CFU of wild-type, *loiA* mutant, or SPI-14 mutant. Data presented are the combination of two independent experiments, with *P* value determined by log-rank curve comparison test (ns, not significant). (B) Bacterial counts recovered from liver and spleen of the BALB/c mice i.p. infected with wild-type, *loiA* mutant or SPI-14 mutant at day 3 post-infection. Data are combined from two independent experiments. Bars represent mean CFU of all mice, with *P* value determined by the Mann-Whitney U test (ns, not significant).
(TIF)

S5 Fig *loiA* mutant did not confer additional virulence defect in systemic infection in a SPI-1 mutant background. Bacterial counts recovered from liver (A) and spleen (B) of the BALB/c mice i.p. infected with 1×10^4^ CFU of wild-type, *loiA* mutant, SPI-1 mutant or SPI-1/*loiA* double mutant at day 3 post-infection. Data are combined from two independent experiments. Bars represent mean CFU of all mice, with *P* value determined by the Mann-Whitney U test (ns, not significant).
(TIF)

S6 Fig Expression of LoiA was increased under SPI-1-inducing conditions. qRT-PCR analysis of *loiA* gene expression under SPI-1-inducing conditions (low O_2_, high salt) and non-inducing conditions (high O_2_, low salt; control) to late-exponential phase. Data are representative of at least three independent experiments and are presented as mean ±SD. *P* values were determined by student’s t test (^***^P<0.001).
(TIF)

S7 Fig *fnr* mutant did not influence LoiA expression. qRT-PCR analysis of *loiA* gene expression in wild-type and *fnr* mutant. Bacteria were grown in LB medium (0.17 M NaCl) either with low O_2_ (A) or high O_2_ (B). Data are representative of at least three independent experiments and are presented as mean ±SD. *P* values were determined by student’s t test.
(TIF)

S8 Fig ArcA does not bind to *loiA* promoter. EMSAs of *loiA* promoter DNA fragment with purified ArcA-His_6_ protein (0, 0.1, 0.2, 0.4, 0.8 and 1.6 μM). *cydA* promoter is used as a positive control.
(TIF)

S9 Fig Mutation of *arcAB* and *loiA* cannot fully eliminate the induction of *hilD* by low O_2_ concentration. qRT-PCR analysis of *hilD* gene expression in wild-type strain grown with high O_2_, and wild-type, *loiA* mutant, *arcA* mutant, and *arcB* mutant strains grown with low O_2_. *hilD* expression levels are collected from the data of Fig 6A and Fig 7C. *hilD* gene expression level in wild-type strain under high O_2_ concentration was used as a control. Data are representative of at least three independent experiments and are presented as mean ±SD. *P* values were determined by student’s t test (^*^P<0.05; ^***^P<0.001).
(TIF)

S1 Table Strains and plasmids used in this study. (DOCX)

S2 Table Primers used in this study. (DOCX)
