# Correction: Signal transduction pathway mediated by the novel regulator LoiA for low oxygen tension induced *Salmonella* Typhimurium invasion

**DOI:** 10.1371/journal.ppat.1007997

**Published:** 2019-08-12

**Authors:** Lingyan Jiang, Lu Feng, Bin Yang, Wenwen Zhang, Peisheng Wang, Xiaohan Jiang, Lei Wang

The authors would like to correct Figs 3 and 7, and S4 Fig because the same data sets were reported in multiple figures. The ΔSPI-14 data should be removed from Fig 3B, 3C and 3D, S4A and S4B Fig. Additionally, the ΔloiA data should be removed from Fig 7E. The authors have provided the correct Fig 3, S4 Fig, and Fig 7 files here.

**Fig 3:**
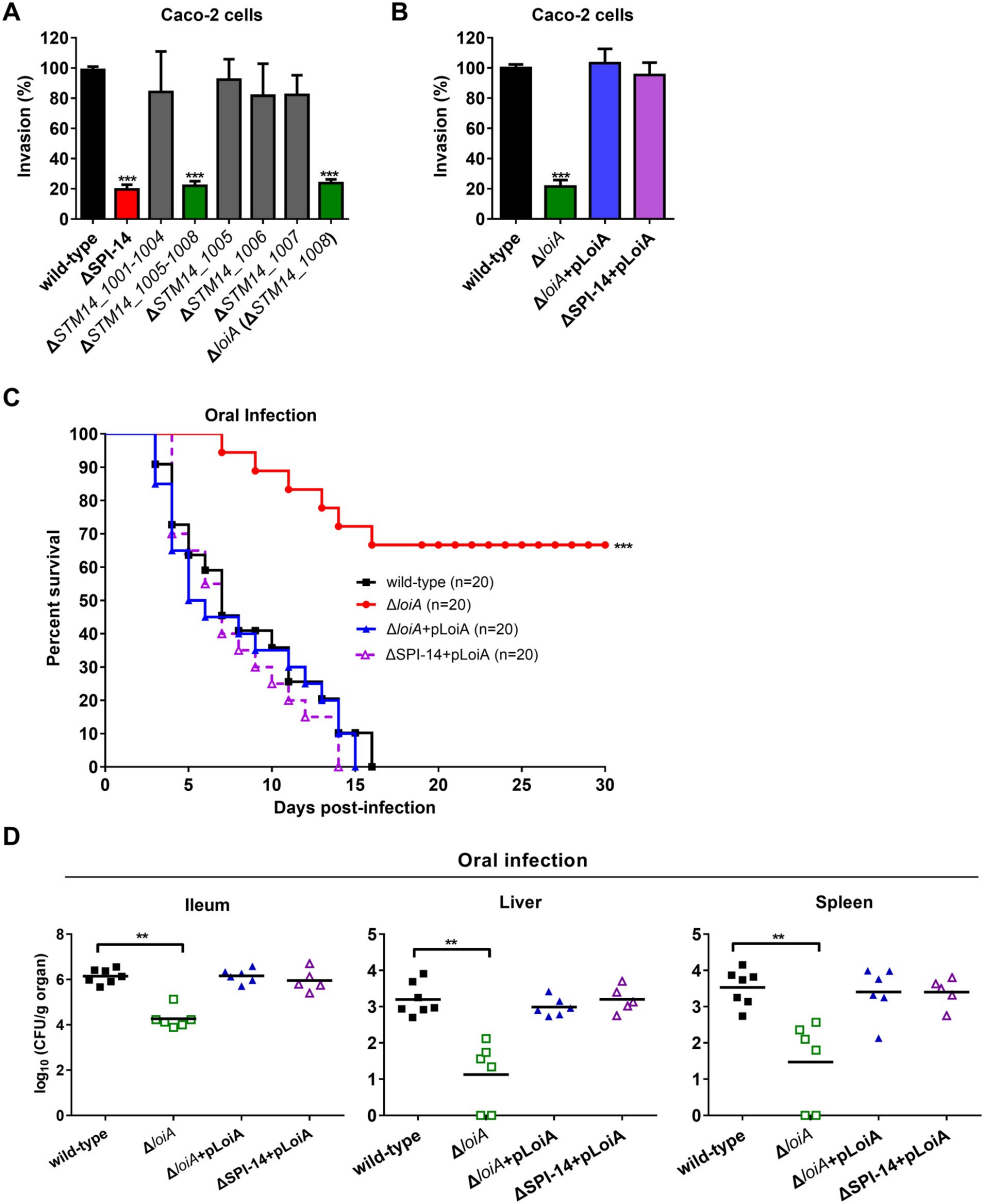
The gene *loiA* (*STM14_1008*) is the virulence determinant in SPI-14 influencing *S*. Typhimurium invasion. (A) Invasion assays of wild-type, *SPI-14* mutant, *STM14_1001-STM14_1004* mutant, *STM14_1005-STM14_1008* mutant, *STM14_1005* mutant, *STM14_1006* mutant, *STM14_1007* mutant and *loiA* (*STM14_1008*) mutant. (B) Invasion assays of wild-type, *loiA* (*STM14_1008*) mutant and complemented strains. The wild-type control is the same as that of Fig 2B, which was obtained from the same batch of experiments. For (A) and (B), Caco-2 cells were infected with bacteria at an MOI of 10. The invasion ability of mutants is reported as percentages relative to the wild-type strain. Data are representative of at least three independent experiments and are presented as mean ±SD. *P* values were determined by student’s t test (***P<0.001). (C) Survival plots of BALB/c mice over a 30-day period after orally infected with ~5×10^6^ CFU of indicated bacterial strains. The wild-type control is the same as that of Fig 1A, which was obtained from the same batch of experiments. Data presented are the combination of three independent experiments, ***P<0.001 by log-rank curve comparison test. (D) Bacterial counts recovered from ileum, liver and spleen of the orally infected mice. At day 5 post-infection, mice organs were harvested and homogenized for colony enumeration. The wild-type control is the same as that of Fig 1B, which was obtained from the same batch of experiments. Data are combined from three independent experiments. Bars represent mean CFU of all mice, with P value determined by the Mann-Whitney U test (**P<0.01).

**Fig 7:**
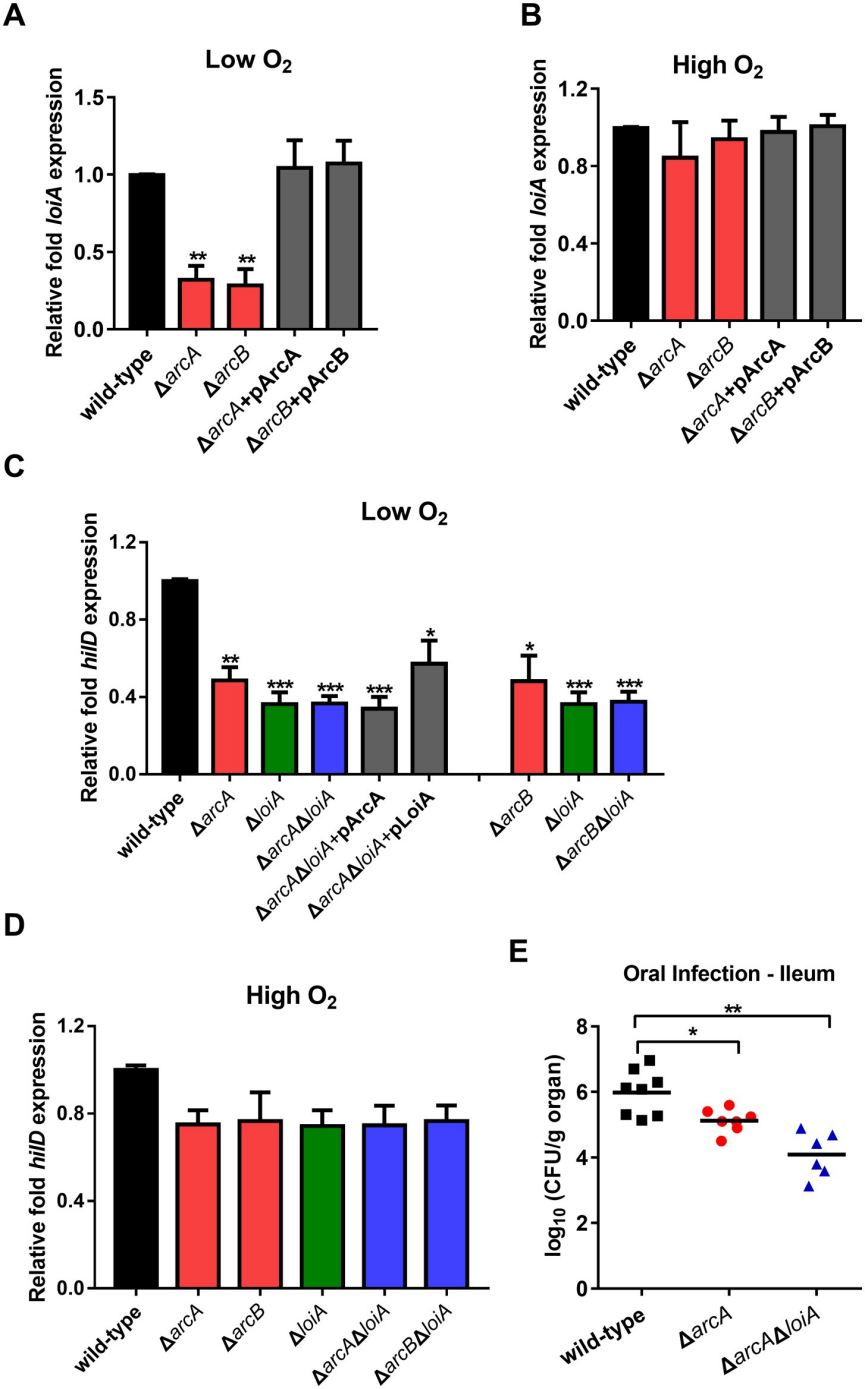
The activation of *LoiA* gene function by low O_2_ conditions is mediated by ArcAB. (A, B) qRT-PCR analysis of *loiA* gene expression in wild-type, *arcA* mutant, *arcB* mutant, and complemented strains for ArcA and ArcB. Bacteria were grown in LB medium (0.17 M NaCl) either with low O_2_ (A) or high O_2_ (B). (C, D) qRT-PCR analysis of *hilD* gene expression in wild-type, *arcA* mutant, *arcB* mutant, *loiA* mutant, *arcA*/*loiA* double mutant, *arcB*/*loiA* double mutant or complemented strains. Bacteria were grown in LB medium (0.17 M NaCl) either with low O_2_ (C) or high O_2_ (D). Data from graphs (A) to (D) are representative of at least three independent experiments and are presented as mean ±SD. *P* values were determined by student’s t test (*P<0.05; **P<0.01). (E) Bacterial counts recovered from ileum of the BALB/c mice orally infected with 5×10^6^ CFU of wild-type, *arcA* mutant or *arcA*/l*oiA* double mutant at day 5 post-infection. The wild-type control is the same as that of Fig 4B (ileum), which was obtained from the same batch of experiments. Data are combined from three independent experiments. Bars represent mean CFU of all mice, with significance determined by the Mann-Whitney U test (*P<0.05; **P<0.01; ns, not significant).

There are errors in the captions for Figs 3 and 7, and S4 Fig. The authors have provided the complete, correct captions for Figs 3 and 7, and S4 Fig here.

## Supporting information

S4 Fig Lack of *loiA* did not influence *S*. Typhimurium systemic infection of BALB/c mice. (A) Survival plots of BALB/c mice after inoculation intraperitoneally (i.p.) with 1×10^4^ CFU of *loiA* mutant. The wild-type control is the same as that of Fig 1C, which was obtained from the same batch of experiments. Data presented are the combination of two independent experiments, with *P* value determined by log-rank curve comparison test (ns, not significant). (B) Bacterial counts recovered from liver and spleen of the BALB/c mice i.p. infected with *loiA* mutant at day 3 post-infection. The wild-type control is the same as that of Fig 1D, which was obtained from the same batch of experiments. Data are combined from two independent experiments. Bars represent mean CFU of all mice, with *P* value determined by the Mann-Whitney U test (ns, not significant).
(TIF)
