# Supplementary figures and images for: Signal transduction pathway mediated by the novel regulator LoiA for low oxygen tension induced Salmonella Typhimurium invasion
Source: PLoS Pathog. 2017 Jun 2;13(6):e1006429. doi: 10.1371/journal.ppat.1006429 (PMC5476282; doi:10.1371/journal.ppat.1006429)

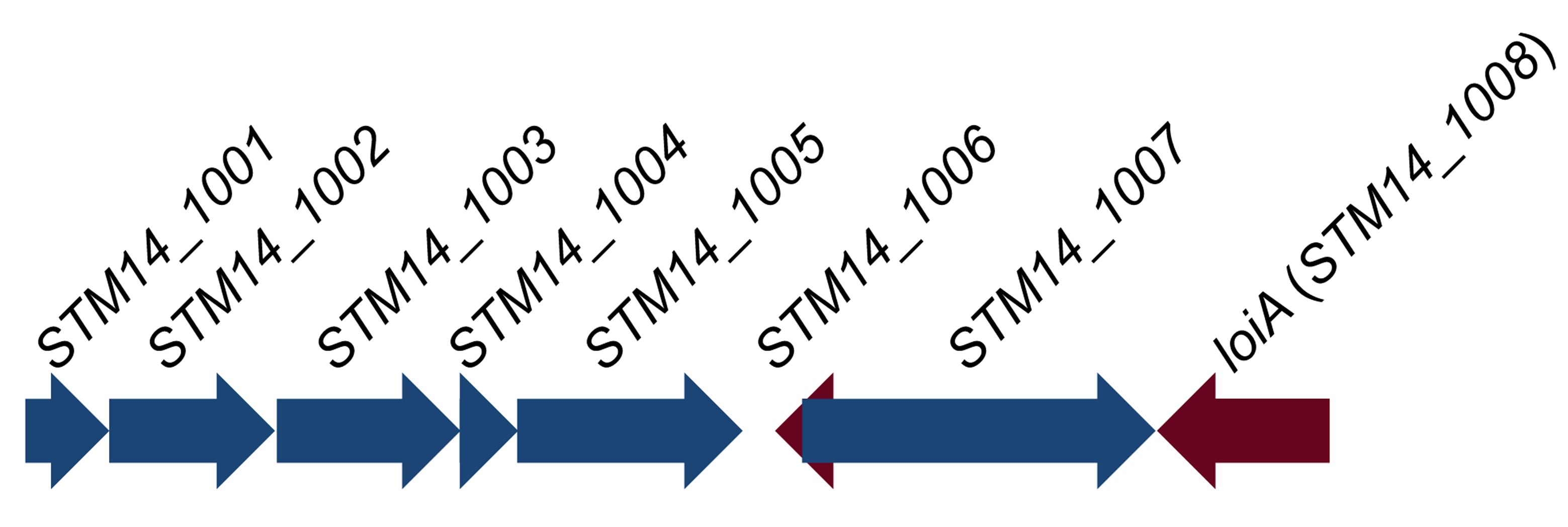

Supplement: S1 Fig — (TIF) [file ppat.1006429.s001.tif]

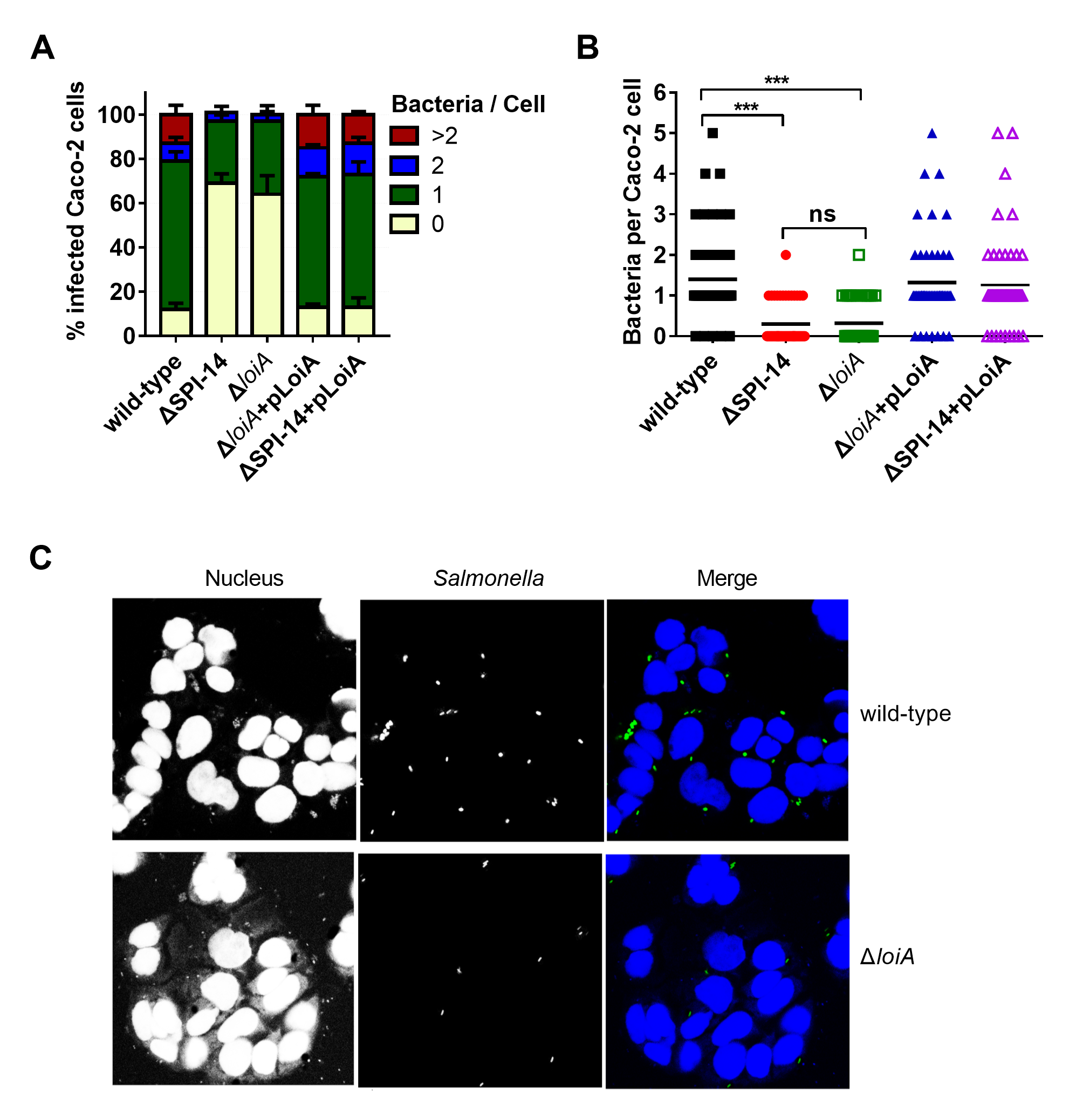

Supplement: S2 Fig — (A) The percentages of infected cells containing 0, 1, 2 or >2 bacteria. Caco-2 cells were seeded on coverslips and infected with bacteria at an MOI of 10. Infected cells were fixed 1 h post-infection and stained for immunofluorescence microscopy. The number of bacteria per Caco-2 cell was counted for at least 50 cells. Data are representative of at least three independent experiments and are presented as mean ±SD. (B) Number of intracellular bacteria per Caco-2 cell. Intracellular bacteria per cell were counted in random fields at 1 h post-infection. Bars show the mean number of bacteria contained in the infected Caco-2 cells that were counted. Data are representative of three independent experiments, with P values determined by student’s t test (***P<0.001; ns, not significant). (C) Representative images of infected Caco-2 cells by wild-type strain and loiA mutant. Bacteria were labelled with anti-Salmonella LPS antibody (green), and cell nuclei were counterstained with DAPI (blue). (TIF) [file ppat.1006429.s002.tif]

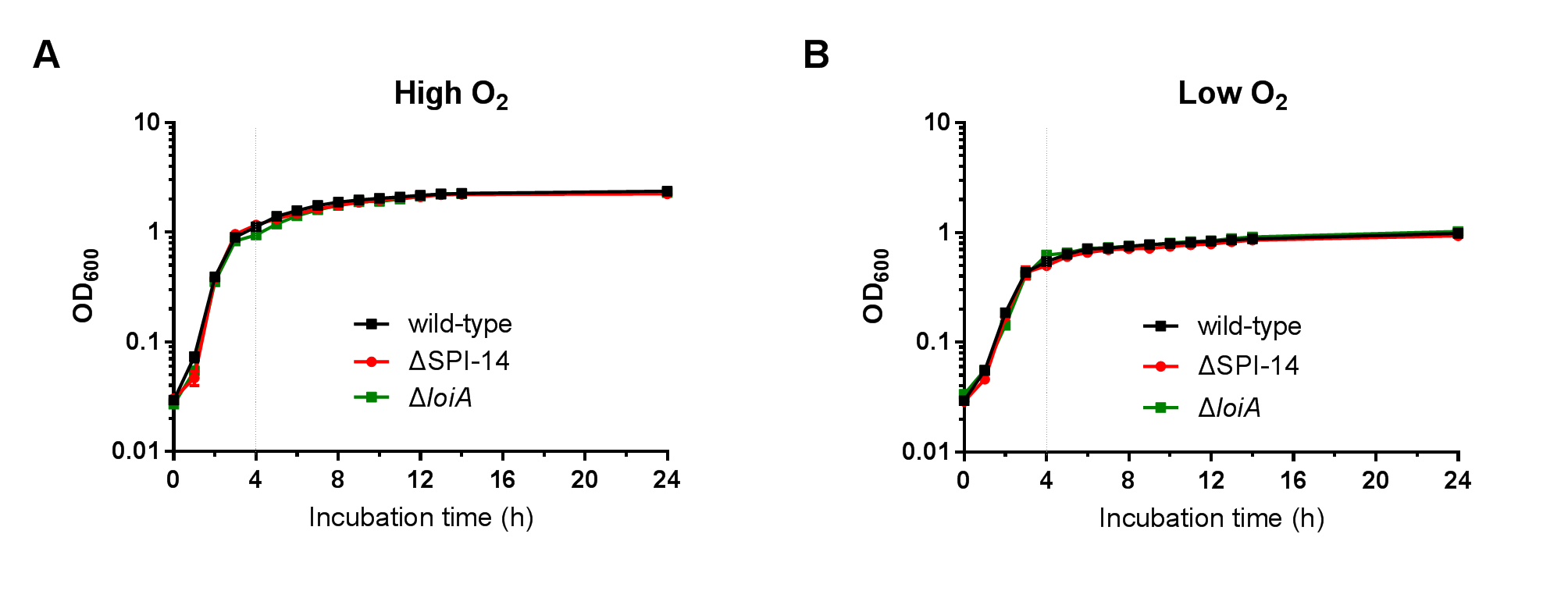

Supplement: S3 Fig — (A, B) Overnight culture of wild-type, SPI-14 mutant or loiA mutant were sub-cultured 1:100 into fresh high salt (0.3 M NaCl) LB medium and cultured for additional 24 h at 37°C with high O2 (A) or low O2 concentrations (B). The absorbance at 600 nm (OD600) of 2 ml aliquots of culture was measured regularly over this period. Data are representative of at least three independent experiments and are presented as mean ±SD. (TIF) [file ppat.1006429.s003.tif]

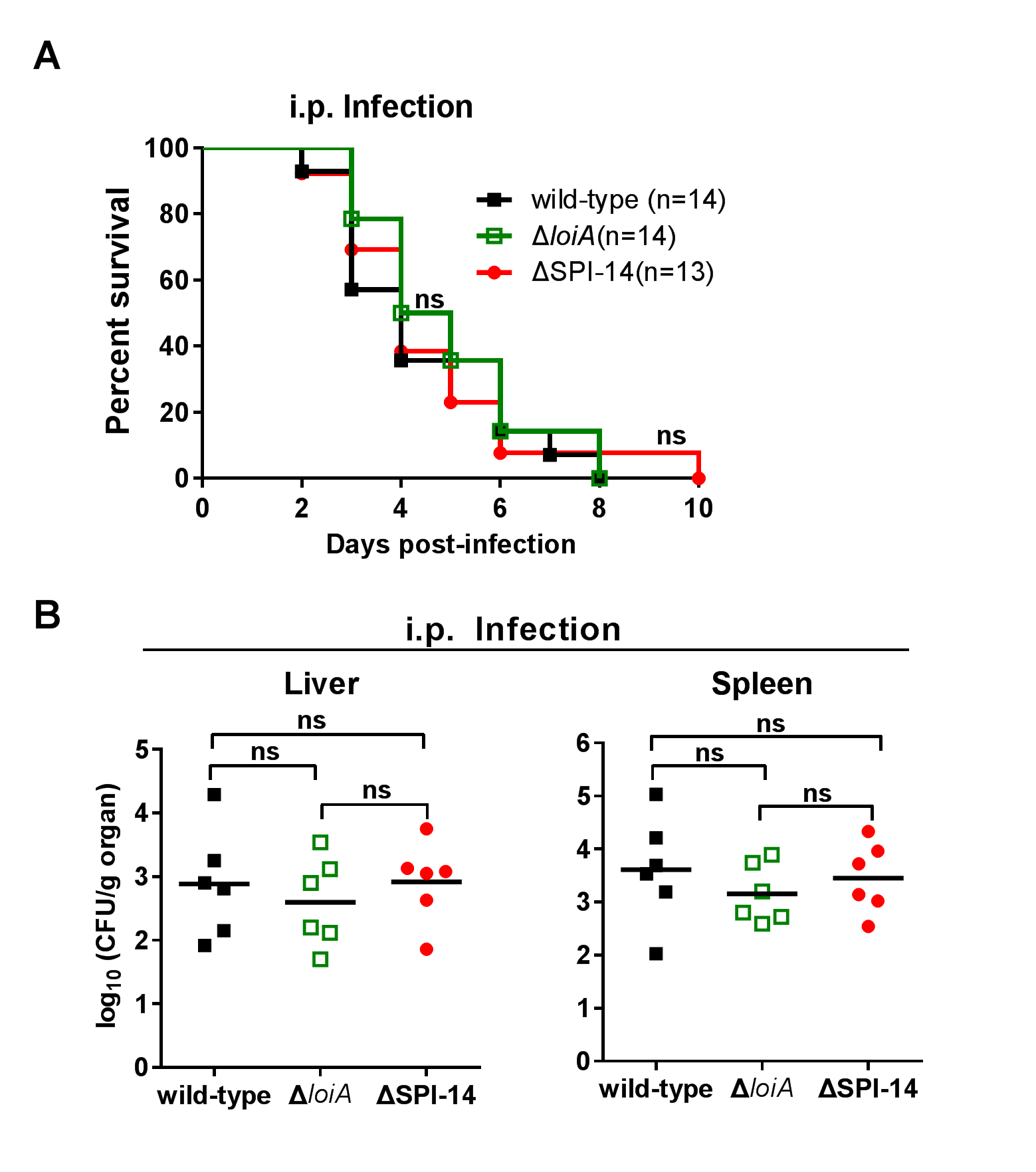

Supplement: S4 Fig — (A) Survival plots of BALB/c mice after inoculation intraperitoneally (i.p.) with 1×10^4^ CFU of wild-type, loiA mutant, or SPI-14 mutant. Data presented are the combination of two independent experiments, with P value determined by log-rank curve comparison test (ns, not significant). (B) Bacterial counts recovered from liver and spleen of the BALB/c mice i.p. infected with wild-type, loiA mutant or SPI-14 mutant at day 3 post-infection. Data are combined from two independent experiments. Bars represent mean CFU of all mice, with P value determined by the Mann-Whitney U test (ns, not significant). (TIF) [file ppat.1006429.s004.tif]

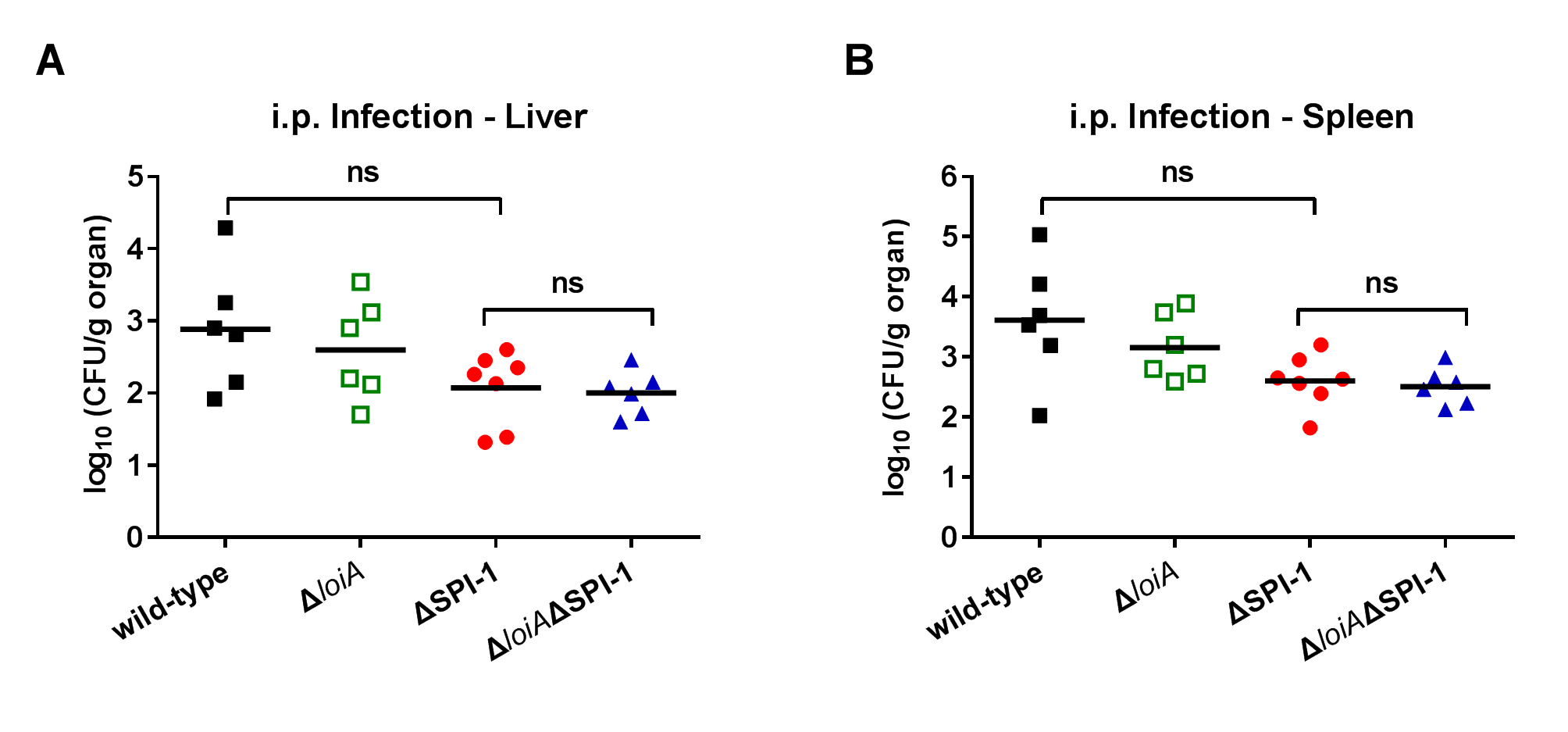

Supplement: S5 Fig — Bacterial counts recovered from liver (A) and spleen (B) of the BALB/c mice i.p. infected with 1×10^4^ CFU of wild-type, loiA mutant, SPI-1 mutant or SPI-1/loiA double mutant at day 3 post-infection. Data are combined from two independent experiments. Bars represent mean CFU of all mice, with P value determined by the Mann-Whitney U test (ns, not significant). (TIF) [file ppat.1006429.s005.tif]

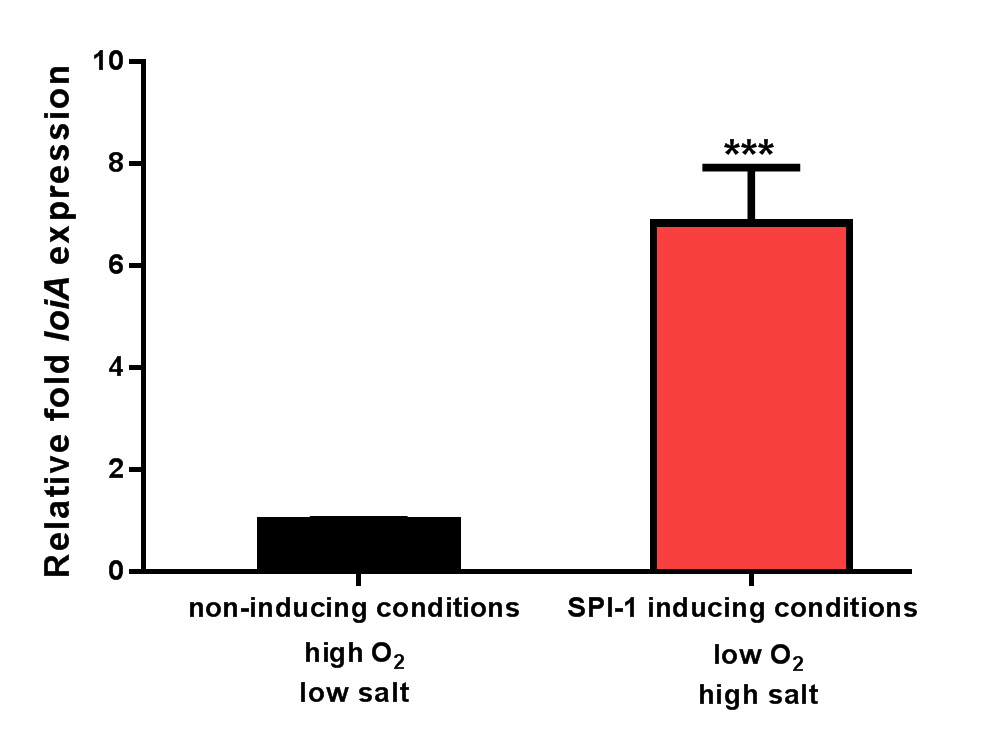

Supplement: S6 Fig — qRT-PCR analysis of loiA gene expression under SPI-1-inducing conditions (low O2, high salt) and non-inducing conditions (high O2, low salt; control) to late-exponential phase. Data are representative of at least three independent experiments and are presented as mean ±SD. P values were determined by student’s t test (***P<0.001). (TIF) [file ppat.1006429.s006.tif]

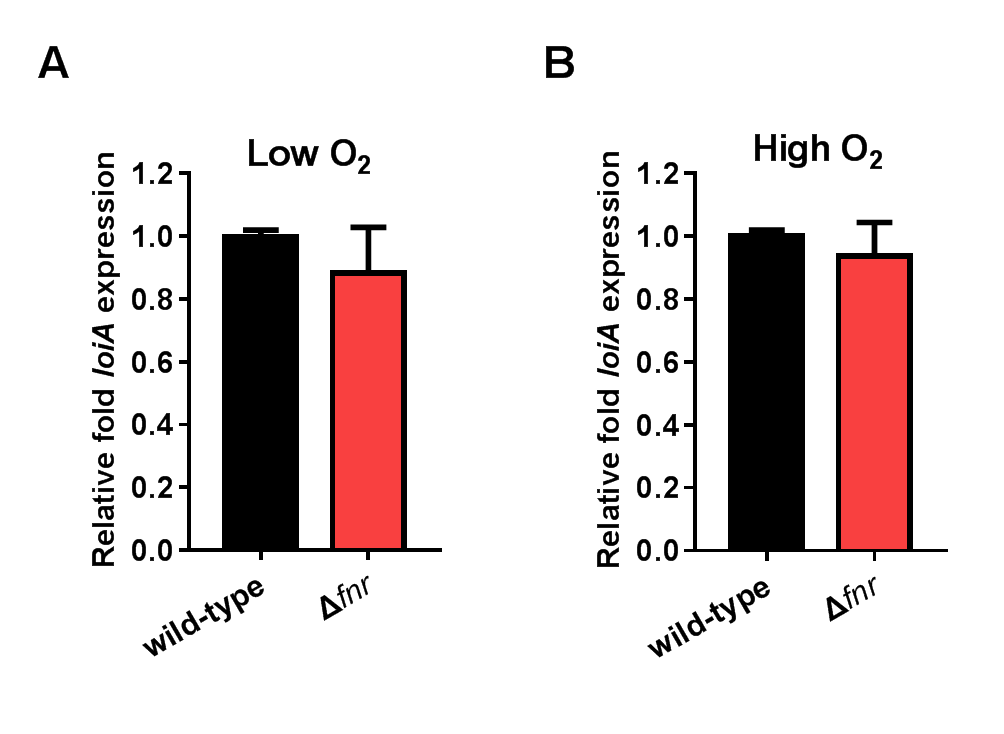

Supplement: S7 Fig — qRT-PCR analysis of loiA gene expression in wild-type and fnr mutant. Bacteria were grown in LB medium (0.17 M NaCl) either with low O2 (A) or high O2 (B). Data are representative of at least three independent experiments and are presented as mean ±SD. P values were determined by student’s t test. (TIF) [file ppat.1006429.s007.tif]

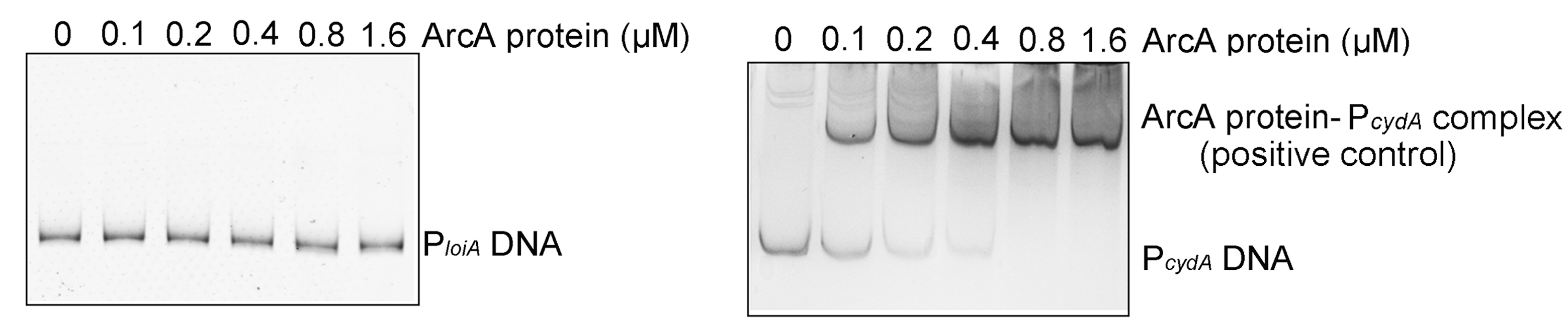

Supplement: S8 Fig — EMSAs of loiA promoter DNA fragment with purified ArcA-His6 protein (0, 0.1, 0.2, 0.4, 0.8 and 1.6 μM). cydA promoter is used as a positive control. (TIF) [file ppat.1006429.s008.tif]

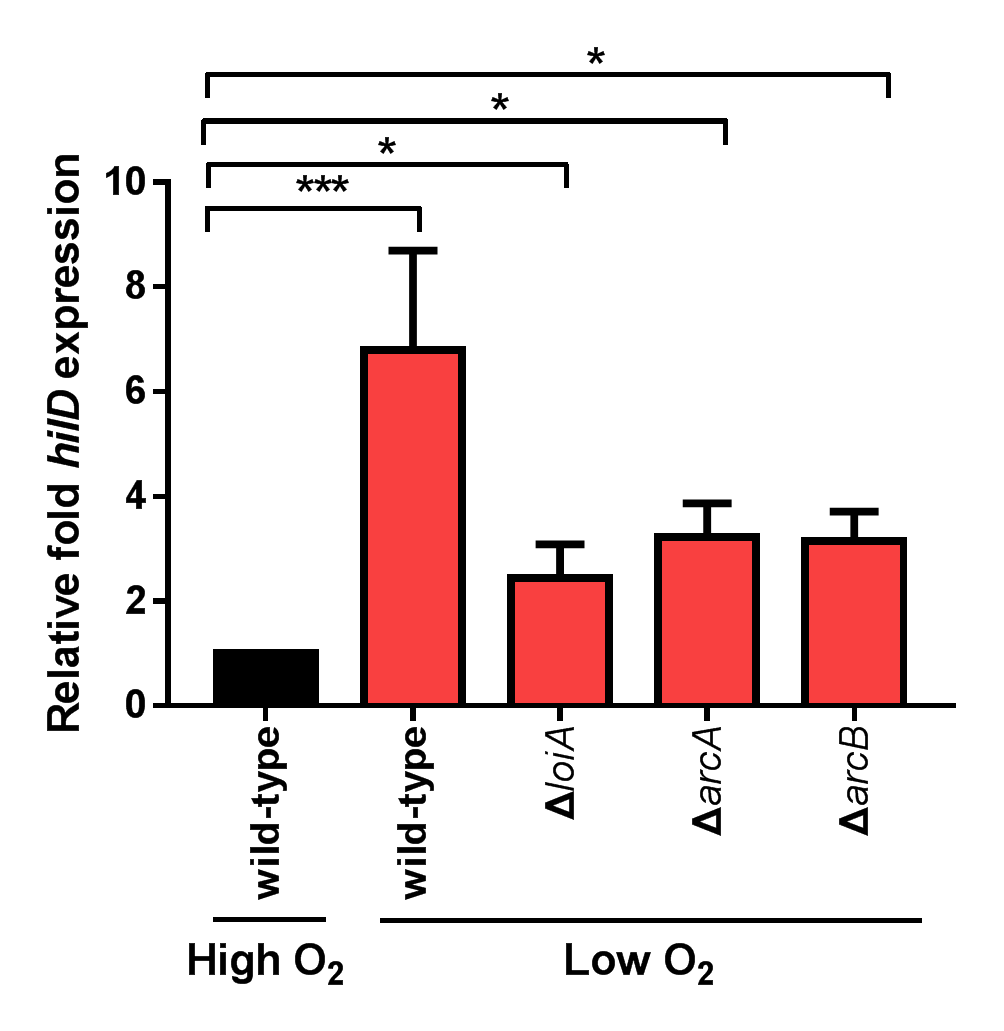

Supplement: S9 Fig — qRT-PCR analysis of hilD gene expression in wild-type strain grown with high O2, and wild-type, loiA mutant, arcA mutant, and arcB mutant strains grown with low O2. hilD expression levels are collected from the data of Fig 6A and Fig 7C. hilD gene expression level in wild-type strain under high O2 concentration was used as a control. Data are representative of at least three independent experiments and are presented as mean ±SD. P values were determined by student’s t test (*P<0.05; ***P<0.001). (TIF) [file ppat.1006429.s009.tif]
