# Supplementary material for: Signal transduction pathway mediated by the novel regulator LoiA for low oxygen tension induced Salmonella Typhimurium invasion
Source: PLoS Pathog. 2017 Jun 2;13(6):e1006429. doi: 10.1371/journal.ppat.1006429 (PMC5476282; doi:10.1371/journal.ppat.1006429)
Supplement: S1 Table — (DOCX) [file ppat.1006429.s010.docx]

**S1 Table. Strains and plasmids used in this study**

| **Plasmid or strain** | **Genotype or description** | **Source** |
| --- | --- | --- |
| **Plasmids** |  |  |
| pKD46 | Red recombinase system under an arabinose-inducible promoter; Ap^R^ | Lab collection |
| pKD3 | Template plasmid containing the Cm cassette for λ Red recombination; Cm^R^ | Lab collection |
| pKD4 | Template plasmid containing the Km cassette for λ Red recombination; Km^R^ | Lab collection |
| pCP20 | a temperature-sensitive replicon expressing the FLP gene to remove antibiotic resistance of mutant strains; Ap^R^ | Lab collection |
| pET28a | T7 expression vector; Km^R^ | Lab collection |
| pWSK129 | low-copy-number expression vector; Km^R^ | Lab collection |
| pWSK-3×FLAG | pWSK129 carrying 3×FLAG sequence and Cm cassette; Cm^R^, Km^R^ | Lab construction |
| pLoiA | pWSK129 carrying the 14028 *loiA* gene; Km^R^ | This study |
| pHilD | pWSK129 carrying the 14028 *hilC* gene; Km^R^ | This study |
| pArcB | pWSK129 carrying the 14028 *arcB* gene; Km^R^ | This study |
| pArcA | pWSK129 carrying the 14028 *arcA* gene; Km^R^ | This study |
| pET-LoiA | pET28a carrying the 14028 *loiA* gene; Km^R^ | This study |
| pET-ArcA | pET28a carrying the 14028 *ArcA* gene; Km^R^ | This study |
| **Strains** |  |  |
| wild-type | Wild-type S. Typhimurium strain ATCC 14028 | ATCC**^*^** |
| △SPI-14 | 14028 strain *STM14_1001- STM14_1008* deleted | This study |
| △*STM14_1001-1004* | 14028 strain *STM14_1001- STM14_1004*::Cm; Cm^R^ | This study |
| △*STM14_1005-1008* | 14028 strain *STM14_1005- STM14_1008*::Cm; Cm^R^ | This study |
| △*STM14_1005* | 14028 strain *STM14_1005*::Cm; Cm^R^ | This study |
| △*STM14_1006* | 14028 strain *STM14_1006*::Cm; Cm^R^ | This study |
| △*STM14_1007* | 14028 strain *STM14_1007*::Cm; Cm^R^ | This study |
| △*loiA* | 14028 strain *loiA* (*STM14_1008*) gene deleted | This study |
| △*hilD* | 14028 strain *hilD* gene deleted | This study |
| △SPI-1 | 14028 strain SPI-1::Cm; Cm^R^ | This study |
| △*loiA*△SPI-1 | 14028 strain *loiA* gene deleted and SPI-1::Cm; Cm^R^ | This study |
| △*loiA*△*hilD* | 14028 strain *hilD* and *loiA* genes double deleted | This study |
| △*loiA* +pLoiA | △*loiA* containing plasmid pLoiA; Km^R^ | This study |
| △SPI-14+pLoiA | △*SPI-14* containing plasmid pLoiA; Km^R^ | This study |
| △*loiA*△*hilD* +pLoiA | △*loiA*△*hilD* containing plasmid pLoiA; Km^R^ | This study |
| △*loiA*△*hilD* +pHilD | △*loiA*△*hilD* containing plasmid pHilD; Km^R^ | This study |
| WT *loiA*-FLAG | 14028 strain *loiA* gene tagged with 3×FLAG tag; Cm^R^ | This study |
| WT *hilA*-FLAG | 14028 strain *hilA* gene tagged with 3×FLAG tag; Cm^R^ | This study |
| △*loiA hilA*-FLAG | △*loiA hilA* gene tagged with 3×FLAG tag; Cm^R^ | This study |
| △*hilD hilA*-FLAG | △*hilD hilA* gene tagged with 3×FLAG tag; Cm^R^ | This study |
| △*loiA*△*hilD hilA*-FLAG | △*loiA*△*hilD hilA* gene tagged with 3×FLAG tag; Cm^R^ | This study |
| △*loiA*△*hilD* +pLoiA *hilA*-FLAG | △*loiA*△*hilD +*pLoiA *hilA* gene tagged with 3×FLAG tag; Cm^R^, Km^R^ | This study |
| △*loiA*△*hilD* +pHilD *hilA*-FLAG | △*loiA*△*hilD +*pHilD *hilA* gene tagged with 3×FLAG tag; Cm^R^, Km^R^ | This study |
| △*fnr* | 14028 strain *fnr*::Km; Km^R^ | This study |
| △*arcA* | 14028 strain *arcA* gene deleted | This study |
| △*arcB* | 14028 strain *arcB* gene deleted | This study |
| △*arcA*△*loiA* | 14028 strain *loiA* gene deleted and *arcA*::Cm; Cm^R^ | This study |
| △*arcB*△*loiA* | 14028 strain *loiA* gene deleted and *arcB*::Cm; Cm^R^ | This study |
| △*arcB*+pArcB | △*arcB* containing plasmid pArcB; Km^R^ | This study |
| △*arcA*+pArcA | △*arcB* containing plasmid pArcA; Km^R^ | This study |
| △*arcA*△*loiA*+pLoiA | △*arcA*△*loiA* containing plasmid pLoiA; Cm^R^, Km^R^ | This study |
| △*arcA*△*loiA*+pArcA | △*arcA*△*loiA* containing plasmid pArcA; Cm^R^, Km^R^ | This study |

^*^ ATCC: American Type Culture Collection, Manassas, Virginia, USA.
