# Supplementary material for: Signal transduction pathway mediated by the novel regulator LoiA for low oxygen tension induced Salmonella Typhimurium invasion
Source: PLoS Pathog. 2017 Jun 2;13(6):e1006429. doi: 10.1371/journal.ppat.1006429 (PMC5476282; doi:10.1371/journal.ppat.1006429)
Supplement: S2 Table — (DOCX) [file ppat.1006429.s011.docx]

**S2 Table. Primers used in this study**

| **Target gene** |  | **Primer sequence (5'-3')** |
| --- | --- | --- |
| ***Primers for construction of mutants and 3×FLAG-tagged strain ^*^*** | | |
| SPI-14 | F | ATGCAAACGTTAGAACGGTTTTTTCTTTTCGTTACGGG GTGTAGGCTGGAGCTGCTTCG |
|  | R | ATGCACTTTGATATAAAAGATTTAAAATTATTCCTTGCT CATATGAATATCCTCCTTAG |
| *STM14_1001- STM14_1004* | F | ATGCAAACGTTAGAACGGTTTTTTCTTTTCGTTACGGG GTGTAGGCTGGAGCTGCTTCG |
|  | R | TCATAATTTTTTCCTCAATAAACCAGCGCCAACAGGGG CATATGAATATCCTCCTTAG |
| *STM14_1005- STM14_1008* | F | ATGAAAAGTATTGAAAAGGATAAATATCAGGATATTCG GTGTAGGCTGGAGCTGCTTCG |
|  | R | ATGCACTTTGATATAAAAGATTTAAAATTATTCCTTGC CATATGAATATCCTCCTTAG |
| *STM14_1005* | F | ATGAAAAGTATTGAAAAGGATAAATATCAGGATATTCG GTGTAGGCTGGAGCTGCTTCG |
|  | R | TCAGAATGAACGTGGCATCTCCAGCACATGCTCAGCGA CATATGAATATCCTCCTTAG |
| *STM14_1006* | F | GTGCCTATAAGACTATATAGCCAGTTATCAACTATTAA GTGTAGGCTGGAGCTGCTTCG |
|  | R | TTATGATGAACGGCTTAAAAATGATTTGTGTATATTCT CATATGAATATCCTCCTTAG |
| *STM14_1007* | F | ATGAACGATACACTTTCTAATACCCAGCAATCACGTGA GTGTAGGCTGGAGCTGCTTCG |
|  | R | TTACATCCCTGTATAATTTGGTCCGTTGCCACCTTCAGGC CATATGAATATCCTCCTTAG |
| *loiA* (*STM14_1008*) | F | ATGCACTTTGATATAAAAGATTTAAAATTATTCCTTGC GTGTAGGCTGGAGCTGCTTCG |
|  | R | TCACTCAGTCTGAGCTGTGAGCATAGTAACCAGTTTTTG CATATGAATATCCTCCTTAG |
| *hilD* | F | ATGGAAAATGTAACCTTTGTAAGTAATAGTCATCAGCGT GTGTAGGCTGGAGCTGCTTCG |
|  | R | TTAATGGTTCGCCATTTTTATGAATGTCGATGGCGTAG CATATGAATATCCTCCTTAG |
| SPI-1 | F | ACAGGCGTAATGAAATCGTACCAGAGGCGCAAGGTTAA GTGTAGGCTGGAGCTGCTTCG |
|  | R | AGTAAAAAGACAGGAAGACAGCTATAAAATTTTTTCAT CATATGAATATCCTCCTTAG |
| *fnr* | F | ATGATCCCGGAAAAGCGAATTATACGGCGCATTCAGTC GTGTAGGCTGGAGCTGCTTCG |
|  | R | TTAAGCGACGTTGCGGGTATGACCGGCGAGGGCCGCCAG CATATGAATATCCTCCTTAG |
| *arcB* | F | ATGAAGCAAATTCGTATGCTGGCGCAATACTATGTCGA GTGTAGGCTGGAGCTGCTTCG |
|  | R | TCATTTTTTTTCCGCGTTTGCCACCCAGGCTTTCAGCAC CATATGAATATCCTCCTTAG |
| *arcA* | F | ATGCAGACCCCGCACATTCTTATCGTTGAAGACGAGTT GTGTAGGCTGGAGCTGCTTCG |
|  | R | TTAATCCTGCAGGTCGCCGCAGAAGCGATAACCTTCGCC CATATGAATATCCTCCTTAG |
| *loiA -3×FLAG* | F | CACAAAAACTGGTTACTATGCTCACAGCTCAGACTGAG GACTACAAAGACCATGACGGTG |
|  | R | AGGTGGCAACGGACCAAATTATACAGGGATGTAACGCTA TTACGCCCCGCCCTGCCACTCA |
| *hilA-3×FLAG* | F | AAAGATGGAAACAGGATCCCCGCTTGATTAAATTACGG GACTACAAAGACCATGACGGTG |
|  | R | CGATGATAAAAAAATAATGCATATCTCCTCTCTCAGATT TTACGCCCCGCCCTGCCACTCA |
| ***Primers for identification of the mutants and 3×FLAG-tagged strain*** | | |
| SPI-14 | F | TTTTCCGCCAAAGGTGACT |
|  | R | TCACCAGCGTCCTACCAGAT |
| *STM14_1001- STM14_1004* | F | TTTTCCGCCAAAGGTGACT |
|  | R | ACGAACCGCATCACGAAT |
| *STM14_1005- STM14_1008* | F | TGTTGGCGCTGGTTTATT |
|  | R | TCACCAGCGTCCTACCAGAT |
| *STM14_1005* | F | TGTTGGCGCTGGTTTATT |
|  | R | GCGAGAAATAATCAGCCAACT |
| *STM14_1006* | F | TGCTCCCATATCTACCAACC |
|  | R | CGTGATTGCTGGGTATTAGA |
| *STM14_1007* | F | GTTGGCTGATTATTTCTCGC |
|  | R | GGTATAATGCCTCACTCTGC |
| *loiA* (*STM14_1008*) | F | TCCGTCTATCTCAGTAACACC |
|  | R | TCACCAGCGTCCTACCAGAT |
| *hilD* | F | AGCAGCAGATTACCGCACAG |
|  | R | CCCATCCTGATAGAGCGTGT |
| SPI-1 | F | TTCTGTCAATCTCACTGCTTAT |
|  | R | CCAACCGTAAAAGTGACCAT |
| *fnr* | F | GGGATAGCTCAGACTTACGC |
|  | R | CATAGCCATACAGGGTCTCC |
| *arcB* | F | ACGATTTCCCTGGTGTTGG |
|  | R | GCTAACGGCAGGTGAGATG |
| *arcA* | F | GACATAAGAAACAGCCAGTAA |
|  | R | GGTTAGGATGACAGCCGTTT |
| *loiA -3×FLAG* | F | TCCGTCTATCTCAGTAACACC |
|  | R | GAAGGGACAGGAATGTTTGA |
| *hilA-3×FLAG* | F | TTCTGGAAAGTGAACAGCGT |
|  | R | TGGGCGATAGCGTAAAGTAG |
| ***Primers for construction of clone and complemented strain*** | | |
| *loiA* | F | GCTCTAGAACGGGTTAACCTGATGACC |
|  | R | CGGGATCCTCACTCAGTCTGAGCTGTG |
| *hilD* | F | GCTCTAGATACCGCACAGGACACAGG |
|  | R | CGGGATCCTTAATGGTTCGCCATTTT |
| *arcB* | F | CGGGATCCCGTGATGAGGGGCGCTA |
|  | R | GGAATTCTCATTTTTTTTCCGCGTT |
| *arcA* | F | CGGGATCCTTTTGACACTGTCGGGTC |
|  | R | GGAATTCTTAATCCTGCAGGTCGCC |
| *loiA-His6* | F | CGCGGATCCAGCCACAACGATGCACTTTG |
|  | R | CCCAAGCTTTCACTCAGTCTGAGCTGTGAGCA |
| *arcA-His6* | F | CGCGGATCCATGCAGACCCCGCACATT |
|  | R | CCCAAGCTTTTAATCCTGCAGGTCGCC |
| ***qRT-PCR primers*** | |  |
| *16S rRNA* | F | GAAAGCGTGGGGAGCAAAC |
|  | R | ACATGCTCCACCGCTTGTG |
| *hilA* | F | CGCTGGCAGAATGCTACCTC |
|  | R | TGTTTGAATAGCAAACTCCCGA |
| *hilC* | F | TTTTCATGCGGACTTGTTGC |
|  | R | CTCAGCCTGTGACCATTTGC |
| *hilD* | F | GCTTTCGGAGCGGTAAACTG |
|  | R | CCAAGTCGTTGCGTCGGTAT |
| *hilE* | F | GCTTACAACCACAACCCGAC |
|  | R | CAGCACGCCTTCTTTCACC |
| *rtsA* | F | TATTACGGCATCAGGGCCA |
|  | R | ACTCTTGCTACGCCTGTTTCTA |
| *sipB* | F | AAGCGACAGAGGCGAAAGAG |
|  | R | AGATTATCCTGCTCACCCTGG |
| *spaO* | F | ACAGAGCGACCGTTTGAGTTG |
|  | R | ATGTGCAACAATTTCCCTTCC |
| *prgK* | F | GCTGAGCCTGATTTTACCGC |
|  | R | AGCCTGCTGACATCACGGA |
| *invF* | F | TGTGCAGCAGAGCGTTGG |
|  | R | TGGGTGATGTTCTCGTGGC |
| *loiA* | F | AAACACCACGCCAGCCTTAT |
|  | R | CGGACTTGGATACTCCCTGAG |
| *invH* | F | TCGGCTGTGCTCAGGTGC |
|  | R | TCTTCGCAAGGTCTGACGG |
| ***Primers for EMSAs and CHIP-qPCR*** | | |
| P*_hilC_* | F | CAATAAGCAGTTTGCGACAT |
|  | R | AGCAAATAATGCAGAAAATGC |
| P*_hilD_* | F | ACTCACAGCCGTTCAGTGAG |
|  | R | CTTCAAGCGTCACGTTAACT |
| P*_loiA_* | F | CTGTTCAAGATTACGTATACG |
|  | R | GGCGTATCATTGTTTGTGG |
| P*_cydA_* | F | ATTACTCTTTGCAAAAACAA |
|  | R | GAACTTGGTCATATCTTTAT |

^*^ Primers were designed carry extensions homologous to 38–40 bp (underlined) of the target gene; F, forward; R, reverse.
