# Supplementary figures and images for: Correction: Signal transduction pathway mediated by the novel regulator LoiA for low oxygen tension induced Salmonella Typhimurium invasion
Source: PLoS Pathog. 2019 Aug 12;15(8):e1007997. doi: 10.1371/journal.ppat.1007997 (PMC6690506; doi:10.1371/journal.ppat.1007997)

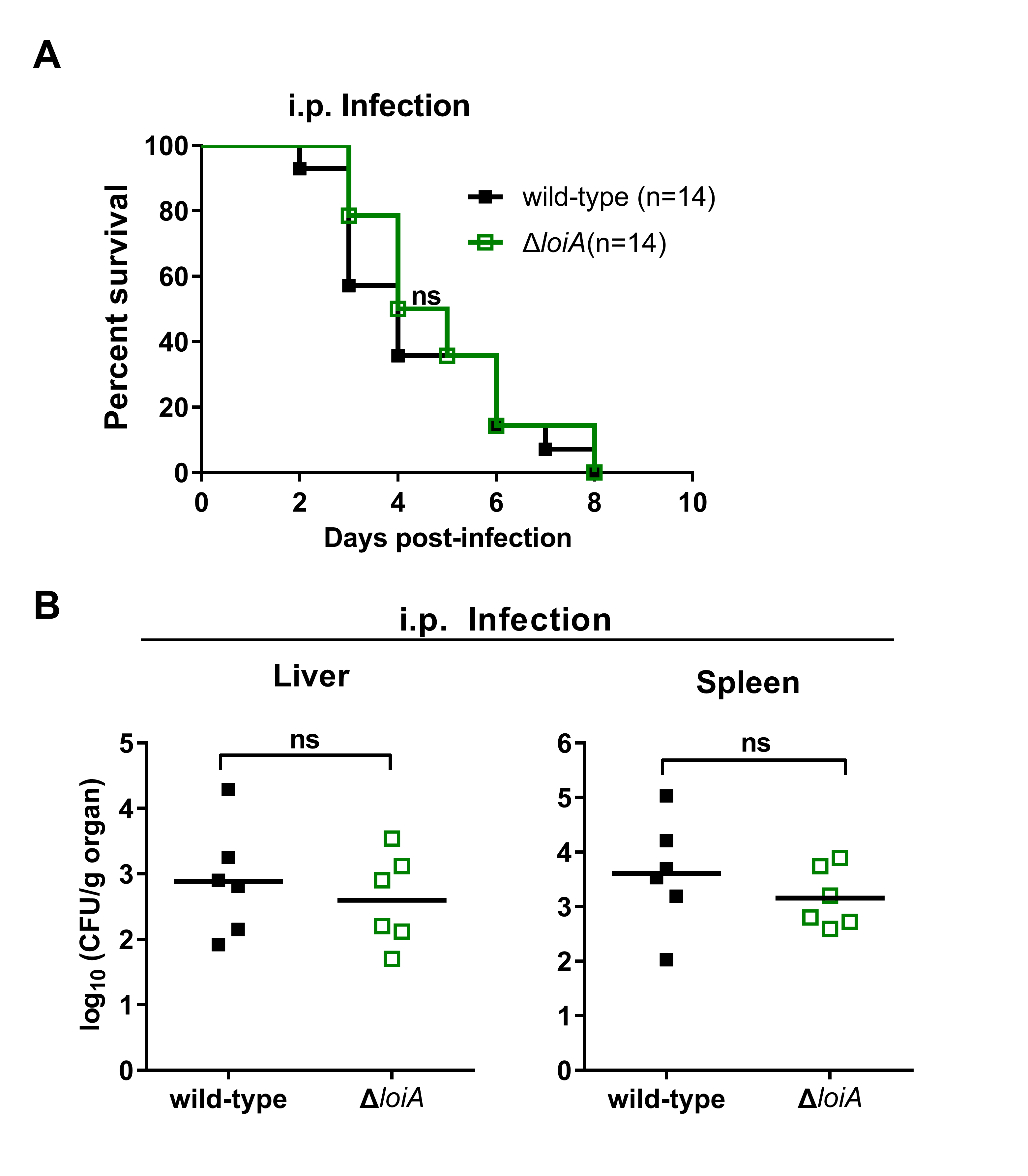

Supplement: S4 Fig — (A) Survival plots of BALB/c mice after inoculation intraperitoneally (i.p.) with 1×10^4^ CFU of loiA mutant. The wild-type control is the same as that of Fig 1C, which was obtained from the same batch of experiments. Data presented are the combination of two independent experiments, with P value determined by log-rank curve comparison test (ns, not significant). (B) Bacterial counts recovered from liver and spleen of the BALB/c mice i.p. infected with loiA mutant at day 3 post-infection. The wild-type control is the same as that of Fig 1D, which was obtained from the same batch of experiments. Data are combined from two independent experiments. Bars represent mean CFU of all mice, with P value determined by the Mann-Whitney U test (ns, not significant). (TIF) [file ppat.1007997.s001.tif]
